# Insights Into Vascular Anomalies, Cancer, and Fibroproliferative Conditions: The Role of Stem Cells and the Renin-Angiotensin System

**DOI:** 10.3389/fsurg.2022.868187

**Published:** 2022-04-27

**Authors:** Ethan J. Kilmister, Swee T. Tan

**Affiliations:** ^1^Gillies McIndoe Research Institute, Wellington, New Zealand; ^2^Wellington Regional Plastic, Maxillofacial & Burns Unit, Hutt Hospital, Lower Hutt, New Zealand; ^3^Department of Surgery, The Royal Melbourne Hospital, The University of Melbourne, Parkville, VIC, Australia

**Keywords:** vascular anomalies, cancer stem cell, fibroproliferative conditions, keloid disorder, Dupuytren's disease, embryonic stem cells, renin-angiotensin system, drug repurposing

## Abstract

Cells exhibiting embryonic stem cell (ESC) characteristics have been demonstrated in vascular anomalies (VAs), cancer, and fibroproliferative conditions, which are commonly managed by plastic surgeons and remain largely unsolved. The efficacy of the mTOR inhibitor sirolimus, and targeted therapies that block the Ras/BRAF/MEK/ERK1/2 and PI3KCA/AKT/mTOR pathways in many types of cancer and VAs, further supports the critical role of ESC-like cells in the pathogenesis of these conditions. ESC-like cells in VAs, cancer, and fibroproliferative conditions express components of the renin-angiotensin system (RAS) – a homeostatic endocrine signaling cascade that regulates cells with ESC characteristics. ESC-like cells are influenced by the Ras/BRAF/MEK/ERK1/2 and PI3KCA/AKT/mTOR pathways, which directly regulate cellular proliferation and stemness, and interact with the RAS at multiple points. Gain-of-function mutations affecting these pathways have been identified in many types of cancer and VAs, that have been treated with targeted therapies with some success. In cancer, the RAS promotes tumor progression, treatment resistance, recurrence, and metastasis. The RAS modulates cellular invasion, migration, proliferation, and angiogenesis. It also indirectly regulates ESC-like cells via its direct influence on the tissue microenvironment and by its interaction with the immune system. *In vitro* studies show that RAS inhibition suppresses the hallmarks of cancer in different experimental models. Numerous epidemiological studies show a reduced incidence of cancer and improved survival outcomes in patients taking RAS inhibitors, although some studies have shown no such effect. The discovery of ESC-like cells that express RAS components in infantile hemangioma (IH) underscores the paradigm shift in the understanding of its programmed biologic behavior and accelerated involution induced by β-blockers and angiotensin-converting enzyme inhibitors. The findings of SOX18 inhibition by R-propranolol suggests the possibility of targeting ESC-like cells in IH without β-adrenergic blockade, and its associated side effects. This article provides an overview of the current knowledge of ESC-like cells and the RAS in VAs, cancer, and fibroproliferative conditions. It also highlights new lines of research and potential novel therapeutic approaches for these unsolved problems in plastic surgery, by targeting the ESC-like cells through manipulation of the RAS, its bypass loops and converging signaling pathways using existing low-cost, commonly available, and safe oral medications.

## Introduction

Plastic surgeons manage an array of disfiguring and life-threatening conditions, many of which remain largely unsolved. An emerging paradigm shift in the understanding of their pathogenesis based on novel concepts, and critical underlying regulatory pathways, offers hope for an effective solution.

There is accumulating evidence for the critical role of cells that possess embryonic stem cell (ESC) characteristics and the expression of components of the renin-angiotensin system (RAS), in different types of vascular anomalies (VAs) (1), cancer (2–6), and fibroproliferative conditions (7–9).

ESCs are cells present in the inner cell mass of human blastocysts, imbued with pluripotency and self-renewal properties (10). Adult stem cells derived from ESCs, such as those in the gastrointestinal tract and skin, are tissue-specific multipotent stem cells that produce a cellular progeny during their life, providing short-lived, specialized cells that perform tissue-specific homeostatic functions (2). Differentiated adult somatic cells can be induced into an ESC state by the introduction of four core transcription factors: octamer-binding transcription factor 4 (OCT4), sex determining region Y-box 2 (SOX2), Krüppel-like factor 4 (KLF4), and c-MYC (11). This can also be achieved by introducing NANOG and LIN28 in place of c-MYC and KLF4 (12). The resultant induced-pluripotent stem cells (iPSCs) that express these stemness-associated markers possess similar pluripotent potential, morphology, gene expression and epigenetic status of genes involved in pluripotency, to ESCs (11).

ESC-like cells that express some or all of the four core transcription factors necessary to convey an iPSC phenotype have been demonstrated in VAs (1) including vascular tumors such as infantile hemangioma (IH) (13) and pyogenic granuloma (14), and vascular malformations such as arterio-venous malformation (AVM) (15), venous malformation (VM) (16), verrucous venous malformation (17), lymphatic malformation (LM) (16), and port-wine stain (PWS) (18). ESC-like cells have also been demonstrated in fibroproliferative conditions such as keloid disorder (KD) (8, 9, 19, 20) and Dupuytren's disease (DD) (21). Similarly, cancer stem cells (CSCs) that express some or all of these stemness-associated markers have been demonstrated in numerous cancer types (22–29), including primary (30) and metastatic (12) head and neck cutaneous squamous cell carcinoma (HNcSCC), and metastatic malignant melanoma (MM) to the head and neck regional nodes (31) and the brain (32).

Components of the RAS have been demonstrated in many cancer types (3–5, 33–35), VAs (1), and fibroproliferative conditions (8, 36), and are localized to cell populations that express stemness-associated markers (1, 3, 8, 37). This article provides an overview of the current knowledge of ESC-like cells and the RAS in these conditions and highlights a new line of research and novel therapeutic strategies.

## The Renin-Angiotensin System

**Figure 1:**
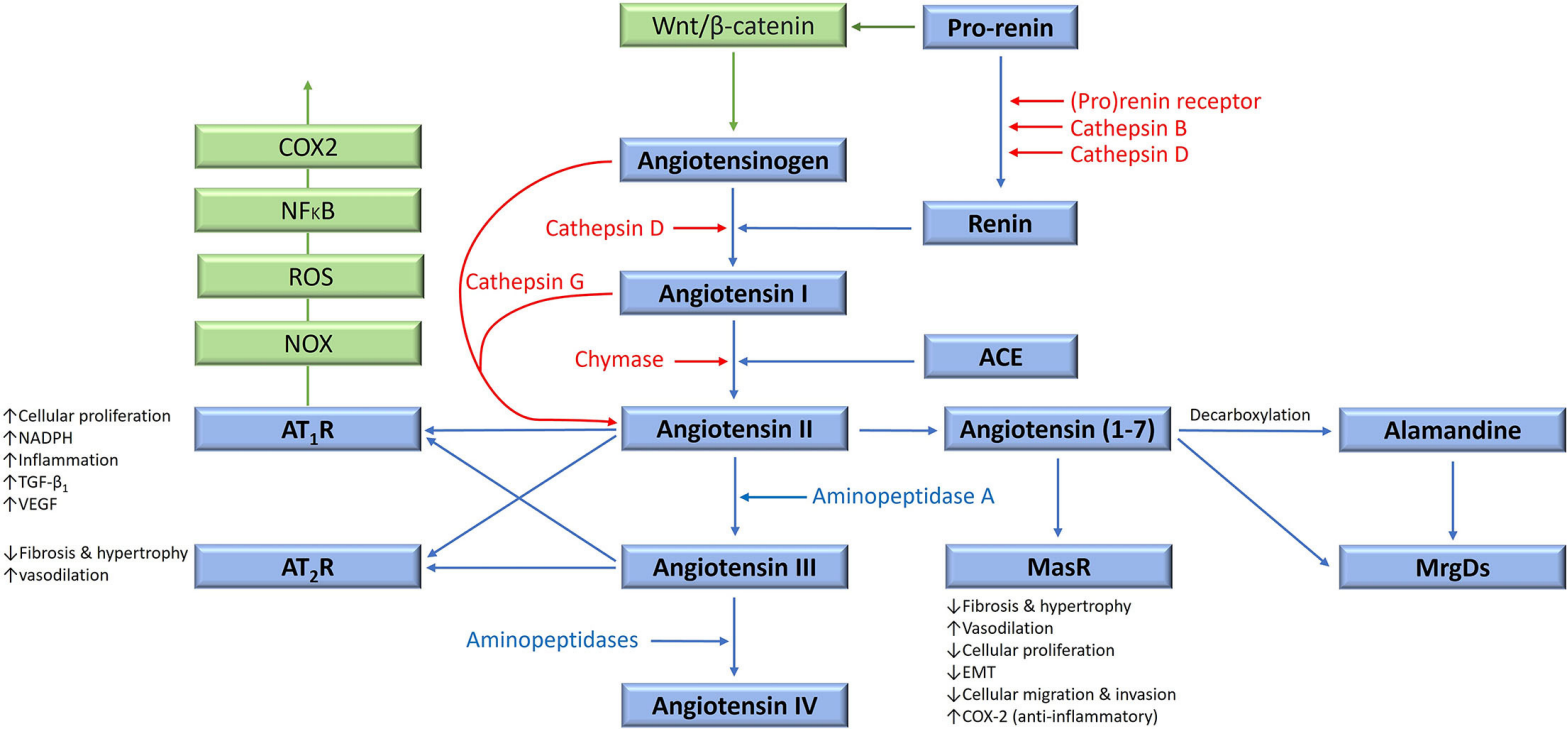
The renin-angiotensin system (RAS), its bypass loops, and converging signaling pathways. In the classical RAS (blue), angiotensinogen is converted to angiotensin I (ATI) by renin. ATI is converted to angiotensin II (ATII) by angiotensin-converting enzyme (ACE). ATII is the main effector for the RAS that binds to ATII receptor 1 (AT_1_R) and ATII receptor 2 (AT_2_R) to exert its physiological effects. Angiotensin 1–7 (Ang1–7) is a cleavage product of ATII which binds to the G protein–coupled receptor Mas receptor (MasR), which in turn exerts physiological effects. Ang1–7 is converted to alamandine by decarboxylation, which acts on Mas-related-G protein coupled receptors (MrgDs). ATII is converted to angiotensin III (ATIII) by aminopeptidase A, and ATIII is converted to angiotensin IV (ATIV) by aminopeptidases. ATII and ATIII bind to both AT_1_R and AT_2_R. Proteases including cathepsins B, D, and G, and chymase constitute bypass loops of the RAS (red), providing redundancies to this system. Other signaling pathways converge on the RAS (green) with the Wnt/β-catenin signaling pathway being upstream of the RAS, activated by (pro)renin receptor. Inflammatory signaling via the NOX-ROS-NFκB-COX2 axis, downstream of the RAS, is induced by activation of AT_1_R. Abbreviations: COX2, cyclooxygenase 2; ROS, reactive oxygen species; VEGF, vascular endothelial growth factor; TGF-β_1_, transforming growth factor β_1_; EMT, epithelial-to-mesenchymal transition; NOX, NADPH oxidase; NFκB, nuclear factor kappa B. *Reproduced and modified with permission from the Journal of Histochemistry and Cytochemistry* (5).

The classical (systemic) RAS is an endocrine cascade resulting in angiotensinogen (AGT) being converted to angiotensin II (ATII) – the physiologically active end-product of the RAS (Figure 1). This system is involved in cardiovascular homeostasis and also operates locally in multiple tissues, including tumors, within the tumor microenvironment (TME).

### Classical Renin-Angiotensin System

Physiologically, AGT is continuously produced by the liver, and is differentially expressed in different tissue types including adipose tissue, heart and kidneys (38). Within the plasma, AGT is converted to angiotensin I (ATI) by renin – a closely regulated enzyme synthesized and released by the juxtaglomerular cells in the kidney. This is a rate limiting step for systemic ATII production (39) (Figure 1).

Renin is produced in an inactive form, which is first cleaved to form pro-renin which is released as an inactive precursor, or is converted by proteases, into intracellular renin which is stored within juxtaglomerular cells (40). Pro-renin binds to the pro-renin receptor (PRR), which involves Wnt signaling, the vacuolar H+ adenosine triphosphatase, Par3 and tyrosine-phosphorylation signaling pathways (41, 42). Renin is then released from granules with in juxtaglomerular cells once stimulated by various mechanisms (39, 43), including the ATII negative feedback (43).

ATI is converted to ATII by angiotensin-converting enzyme (ACE), also known as ACE_1_ – a membrane bound exopeptidase. ACE also degrades vasodilating peptides such as bradykinin, Ang(1-7) and kallikrein, making it an important vasopressor enzyme (39, 44).

ATII exerts its effects via its G-coupled protein receptors – ATII receptor 1 (AT_1_R) and ATII receptor 2 (AT_2_R), resulting in short-term cellular effects, as well as long-term genetic effects (45). AT_1_R signaling also trans-activates epidermal growth factor signaling in breast epithelial cells, resulting in activation of the Ras/RAF/MAPK/ERK pathway, which drives cellular proliferation and cytoskeletal remodeling (46). ATII also causes production of aldosterone, a crucial regulator of blood pressure (47) implicated in oxidative stress and tissue fibrosis (48). Conversely, binding of ATII to AT_2_R opposes the actions of AT_1_R, although much remains unknown about this less investigated ATII receptor (49). Both AT_1_R and AT_2_R are expressed in most human tissues, with AT_1_R being significantly more abundant in adult tissues, with the converse being true during fetal development (49).

ATII is converted to angiotensin III (ATIII), and then angiotensin IV (ATIV), by aminopeptidases (Figure 1). ATIII binds to AT_1_R with less affinity than ATII, and conversely, AT_2_R with more affinity than ATII. ATII is cleaved by angiotensin-converting enzyme 2 to produce Ang(1-7), which then binds to Mas receptors (MasRs). Ang(1-7) can also bind to AT_2_R, and to Mas-related-G protein coupled receptors (MrgDs). Alamandine is the main ligand for MrgDs, which is formed by decarboxylation of Ang(1-7) (50). The Ang(1-7)/MasR axis is protective, by various mechanisms, which include inhibition of cellular proliferation and invasion, metastasis, and epithelial-to-mesenchymal transition (EMT), in several cancer types (51). However, pro-tumorigenic roles of the Ang(1-7)/MasR have been demonstrated in some cancers. For example, Ang(1-7)/MasR signaling stimulates the migration ability of cancer cells in renal cell carcinoma and renal cell adenocarcinoma (52). Interestingly, downstream Ang(1-7)/MasR signaling may be involved in platinum-resistance in non-small cell lung cancer, by inhibiting of angiogenesis and cancer growth (53).

As an endocrine cascade classically involved in the regulation of blood pressure and electrolyte homeostasis, the RAS is traditionally targeted using β-blockers, ACE inhibitors (ACEIs) and angiotensin receptor blockers (ARBs) in the treatment of hypertension and other cardiovascular diseases (54).

### Bypass Loops of the Renin-Angiotensin System

The RAS has multiple bypass loops consisting of lysosomal proteases such as cathepsins B, D and G, and the mast cell protease chymase (Figure 1). Pro-renin is converted to renin by either cathepsin B or cathepsin D. Cathepsin D converts AGT to ATI. Cathepsin G converts AGT directly to ATII, or from ATI to ATII (3). Chymase converts ATI to ATII (55). These bypass loops are relevant, as the effect of traditional RAS inhibitors (RASIs) in blocking the RAS may be circumvented by the activation of these bypass loops, resulting in shunting of precursor peptides through for continued production of the downstream ATII.

### Local Tissue Renin-Angiotensin System and the Tissue Microenvironment

In addition to acting systemically, there is increasing evidence of the RAS acting as a paracrine system in local tissues, as a key homeostatic “gatekeeper” broadly affecting multiple organ systems (38). Components of the RAS have been demonstrated in a wide-range of normal tissues (38), which act independently in an autocrine and paracrine fashion, and/or integrates with the systemic RAS, to exert its endocrine effects (56). A local RAS has been identified in the kidney (57), heart (58, 59), lung (60), brain (50), liver (61), pancreas (62), and is implicated in fibroproliferative and inflammatory conditions in specific organs, independent of the endocrine RAS (63).

A local RAS also operates within the bone marrow (BM) microenvironment, to regulate erythropoiesis, hematopoiesis, myelopoiesis, thrombopoiesis, and the formation of lymphocytic and monocytic lineages (64). The local RAS regulates primitive hematopoiesis in embryonic development, with the first hematopoietic and endothelial cells forming the marrow cavity in the embryo expressing ACE (64). Goker et al. (65) demonstrate that as well as influencing cellular proliferation across a host of tissues including the BM, RAS peptides significantly influence the pluripotential of hematopoietic stem cells. Haznedaroglu et al. (66) demonstrate that ATII utilizes the JAK/STAT pathway, which acts as a point of cross-talk between the local BM RAS and hematopoiesis. The local BM autocrine RAS is implicated in neoplastic hematopoiesis, with multiple RAS components being identified on leukemic blast cells (64). Renin is expressed on cells from acute myeloid leukemia, chronic myeloid leukemia, and acute lymphocytic leukemia (64) and its expression disappears following acute myeloid leukemia remission (67).

By interacting with the immune system, the RAS influences the TME which regulates resident ESC-like cells (5, 35), including CSCs (5). Aberrant RAS signaling is associated with organ dysfunction, and its role in these different groups of diseases is supported by both *in vitro* and *in vivo* studies (1, 3, 8) which highlight its fundamental homeostatic role in cell growth, migration and death, and inflammation (5, 68). Components of the RAS are present and are localized to ESC-like cells in different types of cancer (3–5, 34, 35, 68), VAs (1), and fibroproliferative conditions (8, 36).

### The Role of the Renin-Angiotensin System and the Immune System in the Tumor Microenvironment

The RAS influences the TME largely through its effects on the immune system, in particular, its role in creating immune-suppressive conditions (69). CSCs reside within the TME, which comprises various cellular and non-cellular constituents (70). One of the cellular elements - the endothelial cells, facilitate tumor development and protect tumor cells from the immune system (70). Also, endothelial progenitor cells within the TME contribute to angiogenesis from pre-existing vessels to foster tumor blood supply (71). Immune cells, such as macrophages, lymphocytes, and granulocytes, are another cellular constituent that contribute to an immunosuppressive TME to facilitate tumor cell survival (70). Extra-cellular matrix (ECM) is an essential non-cellular constituent of the TME, which consists of a network of glycoproteins, enzymes, and collagens. ECM also contains critical tissue components that regulate cellular proliferation, adhesion, communication between cells in the TME, and cancer cell migration (70). Further research is needed to understand how endothelial cells, immune cells, and the ECM confer a survival advantage to CSCs, and whether these interactions can be interrupted by inhibiting the RAS, which operates locally within the TME in different cancer types (5, 35). The RAS is expressed by various constituents of the TME, including CSCs, immune cells such as monocytes, macrophages, dendritic cells, neutrophils and T cells, with RAS signaling regulating these cells within the TME (69). The TME can promote or antagonize tumor growth, and modulates apoptosis, angiogenesis, cellular proliferation, metastasis, cancer-associated inflammation and desmoplasia (68, 69). The latter effects are generally driven by the ATII/AT_1_R axis, with these pro-cancer effects being antagonized by the ATII/AT_2_R and Ang(1-7)/MAS axes (69).

The role of the RAS in promoting cancer-associated inflammation and attracting tumor-promoting immune cells which enhance immunosuppression in the TME, is well documented (69). Furthermore, ATII/AT_1_R axis signaling leads to hypoxia and acidosis within the TME, which increases the production of various inflammatory cytokines, transcription factors and the growth factors VEGF, hypoxia-inducible factor, and TGF-β. This creates an immunosuppressive TME, in which there are: (1) impaired function of T and dendritic cells, (2) buildup of immunosuppressive immune cells including myeloid derived suppressor cells, T_reg_ cells, and M2-like macrophages, and (3) elevated expression of PD-L1 and other immune checkpoint molecules that operate in cancer and immune cells (69). Treatment with RASIs may reduce the immunosuppressive effects of the RAS on the TME, which may confer improved treatment outcomes.

Taken together, the RAS – a fundamental homeostatic system – has far reaching effects systemically and locally in many tissue microenvironments, including the TME, with the immune system. Its effect on stem cells is in maintaining normal function of bone marrow stem cells, and in neoplastic hematopoiesis and other hematologic malignancies. We speculate that pathologic processes driven by the RAS may be modulated by RASIs and inhibitors of its bypass loops.

## Embryonic Stem Cell-Like Cells and the Renin-Angiotensin System in Vascular Anomalies

**Figure 2:**
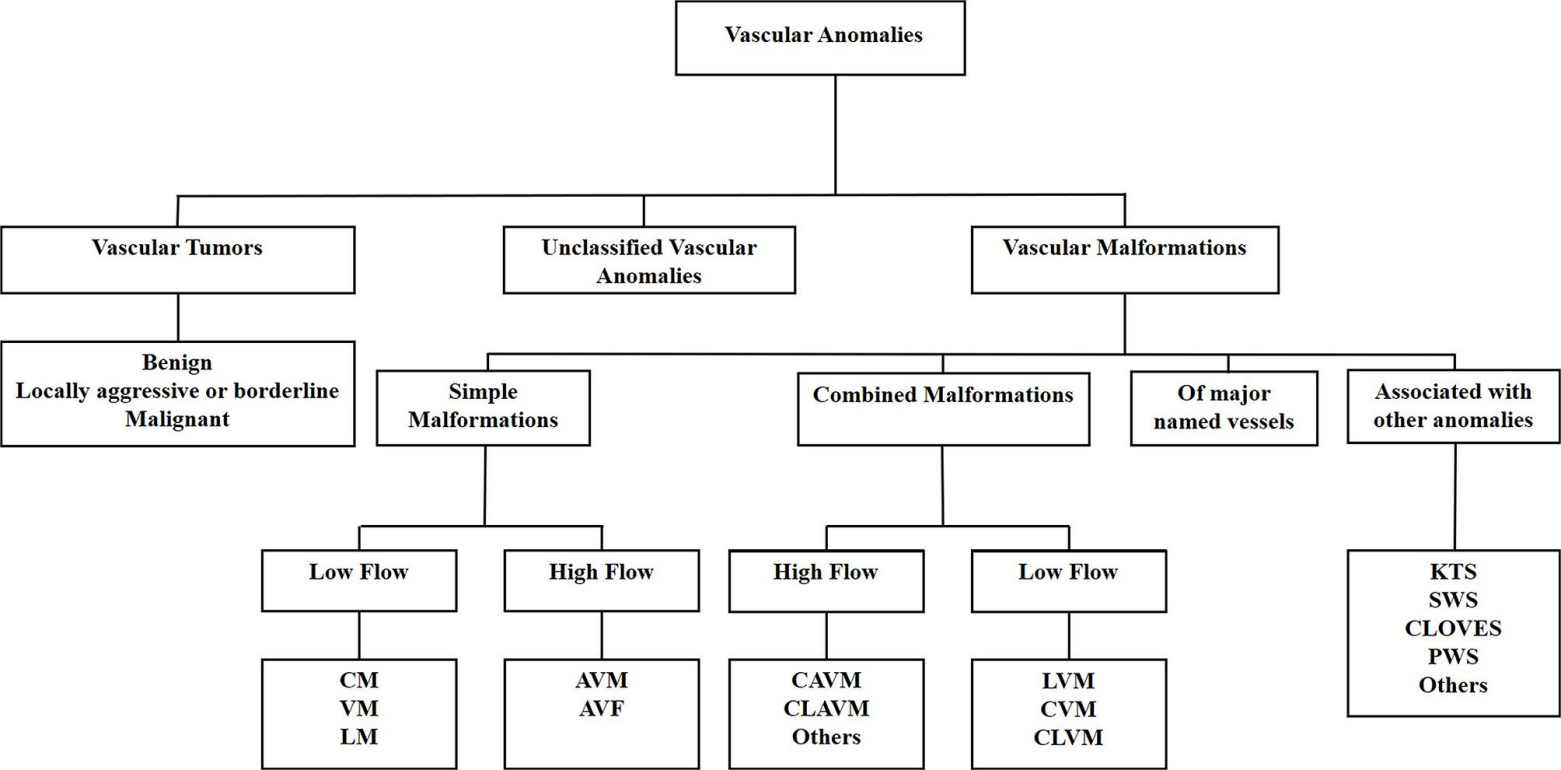
The International Society for the Study of Vascular Anomalies classification of vascular anomalies. Vascular anomalies (VAs) are classified as vascular tumors, vascular malformations and unclassified VAs. Vascular tumors are categorized as being benign, locally aggressive or borderline, or malignant. Vascular malformations are categorized as simple malformations, combined malformations, of major named vessels, or are associated with other anomalies. Simple and combined malformations can be either high-or low-flow. AVF, arterio-venous fistula; AVM, arterio-venous malformation; CAVM, capillary-arteriovenous malformation; CLAVM, capillary-lymphatic-arteriovenous malformation; CLOVES, congenital lipomatous overgrowth-vascular malformation-epidermal nevi-spinal anomaly syndrome; CLVM, capillary-lymphatico-venous malformation; CM, capillary malformation; CVM, capillary-venous malformation; KTS, Klippel-Trénaunay syndrome; LM, lymphatic malformation; LVM, lymphatico-venous malformation; PWS, port-wine stain; SWS, Sturge-Weber syndrome; VM, venous malformation. *Reproduced with permission from Frontiers in Surgery* (1).

VAs are a heterogenous group of conditions, largely treated empirically, often with unsatisfactory outcomes. VAs are classified as vascular tumors or vascular malformations according to the International Society for the Study of Vascular Anomalies classification system (72) (Figure 2). Vascular tumors are categorized as benign, locally aggressive or borderline, and malignant. Vascular malformations are categorized into simple, combined, of major named vessels, and associated with other anomalies. Simple vascular malformations may be further categorized as high-flow or low-flow lesions (73).

Cells expressing stemness-associated markers have been identified in vascular tumors such as IH (74) and pyogenic granuloma (14), and vascular malformations, including VM (16), verrucous venous malformation (17), LM (75), AVM (15), and PWS (18). Components of the RAS are expressed by these ESC-like cells in IH (76), pyogenic granuloma (77), VM (78, 79) and LM (80). This article focuses on the recent paradigm shift in the understanding and treatment of IH and discusses emerging findings of the role of ESC-like cells and the RAS in other VAs.

### A Paradigm Shift in the Understanding and Treatment of Infantile Hemangioma

**Figure 3:**
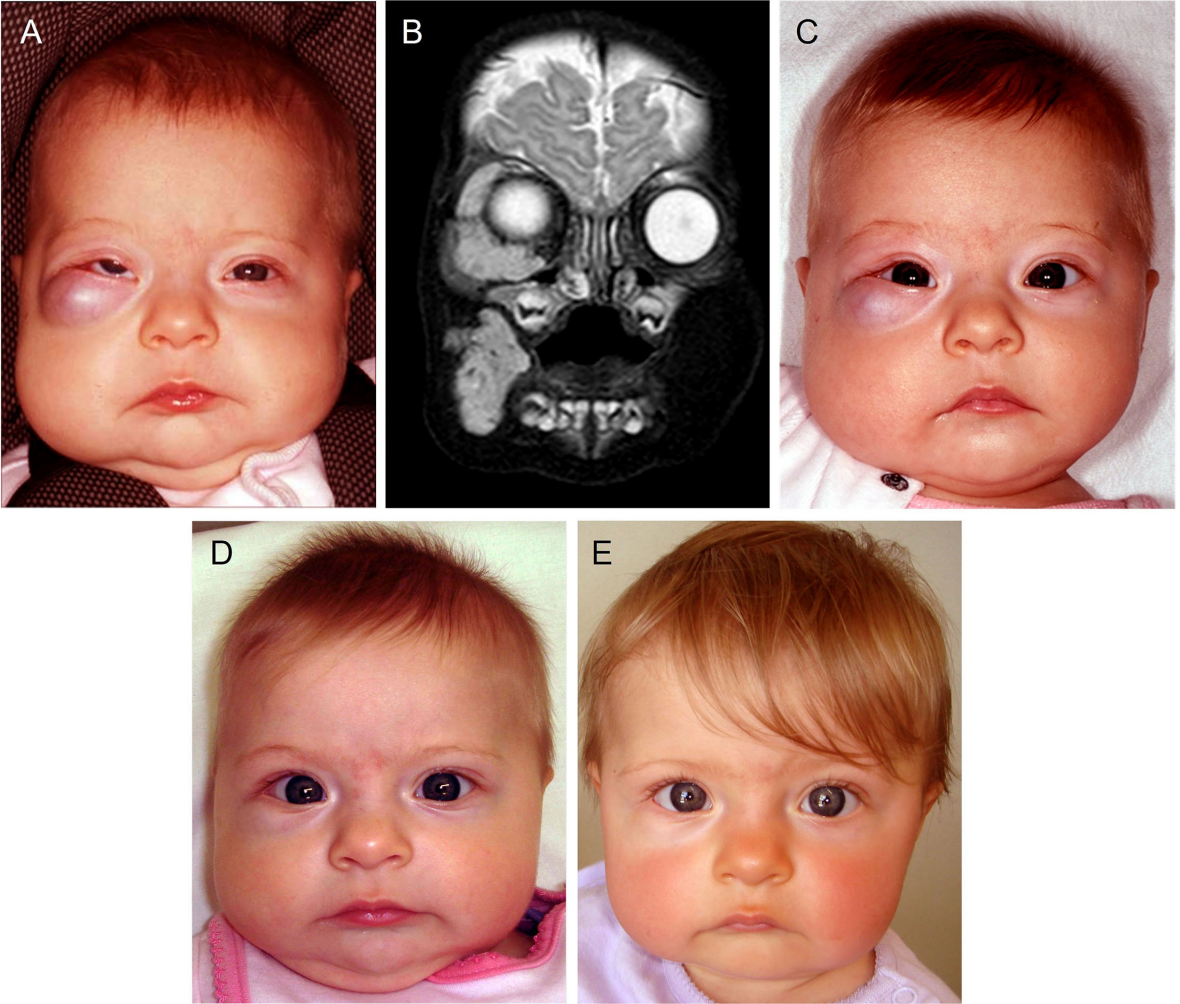
A 4-month-old girl presented with a rapidly growing infantile hemangioma on the right cheek, lower lid and orbit with ocular dystopia **(A)**, as seen on a T_2_-weighted magnetic resonance imaging scan **(B)**. Accelerated involution of the lesion with equalization of the globe **(C)** 7 days, **(D)** 4 weeks and **(E)** 5 months after institution of propranolol therapy at 2mg/kg/day. *Reproduced with permission from Plastic Reconstructive Surgery* (86).

IH, the most common VA, affects 4–10% of infants (13). It is characterized by an initial rapid proliferation followed by spontaneous slow involution for up to 10 years, often leaving a fibrofatty residuum (13). Approximately 15% of IH require intervention during infancy due to a threat to function or life, tissue distortion, or ulceration (81). Problematic proliferating IHs were traditionally treated with high-dose corticosteroids, with interferon and vincristine indicated in refractory cases (82). Accelerated involution of IH induced by propranolol (a non-selective β-blocker) (83) and acebutolol (a selective β_1_-blocker) (84) was serendipitously observed by two independent French groups. This led to a paradigm shift in the treatment of IH, with propranolol now being the mainstay treatment for problematic proliferating IH (Figure 3), although, many other β-blockers have been used (81, 85, 86), indicating a common mode of action by this class of drugs.

The proposed mechanisms of accelerated involution of proliferating IH induced by propranolol include inhibition of angiogenesis, vasoconstriction, and induction of apoptosis of proliferating endothelial cells (85), with mounting evidence implicating the RAS (13, 85, 87).

Research over the last two decades has demonstrated the expression of stemness-associated markers OCT4, stage-specific embryonic antigen-4 (SSEA-4), and STAT-3 on the microvessels of proliferating IH, and a stromal cell population expressing the stemness-associated markers NANOG (74), SALL4 (88), and CD133 (88). The expression of these markers decreases during involution of IH (74). The endothelium of proliferating IH also expresses the primitive mesoderm marker brachyury, hematopoietic markers including ACE (89), neural crest cell and neural crest stem cell markers (90), mesenchymal markers including vimentin and CD29, and the MSC marker Pref-1(13, 91), with a capability of undergoing spontaneous primitive erythropoiesis *in vitro* (13, 92). This phenotype and functional capability suggests the presence of a hemogenic endothelium phenotype (13, 89). Hemogenic endothelium gives rise to progenitor cells and hematopoietic stem cells in the yolk sac, aorta and placenta during embryonic development (93). There is also compelling evidence showing a placental origin of IH with this primitive endothelium sharing the phenotypic signature of placental chorionic villous mesenchymal core cells (13, 94).

The endothelium of proliferating IH expresses components of the RAS: ACE, AT_2_R and PRR (76), with PRR being expressed by both endothelial and non-endothelial cells (95). Renin promotes endothelial cell proliferation in IH which is blocked by dickkopf-1 — a Wnt receptor blocker of Wnt signaling (96). This suggests there are interactions between renin, Wnt signaling, and the PRR (95) which is a part of the Wnt/frizzled receptor complex (97) (Figure 1).

RAS modulation influences cellular proliferation in IH, *in vitro* (1). Treatment of IH explants with an ACEI or AT_2_R antagonist significantly reduces expression of Ki67, a marker of cellular proliferation. AT_2_R agonism increases the proportion of Ki67^+^ cells, and treatment with the ACEI ramipril, prevents ATI-induced cellular proliferation in IH explants (98).

Plasma renin activity is highest in the first year of life, being 17-fold higher compared to adulthood, which then decreases rapidly to 8-fold of adult levels at 1–4 years of age, and then 5-fold at 5–9 years of age, followed by a tapering to normal levels in adults at 10–15 years old (99). The inverse correlation of serum levels of renin activity with age from birth, mirrors the programmed biologic behavior of IH (99–101). The important role of renin in fueling proliferation of IH is further supported by an increased incidence of IH in white, female, and premature infants, all of whom have elevated endogenous levels of renin, compared to their counterparts (87).

**Figure 4:**
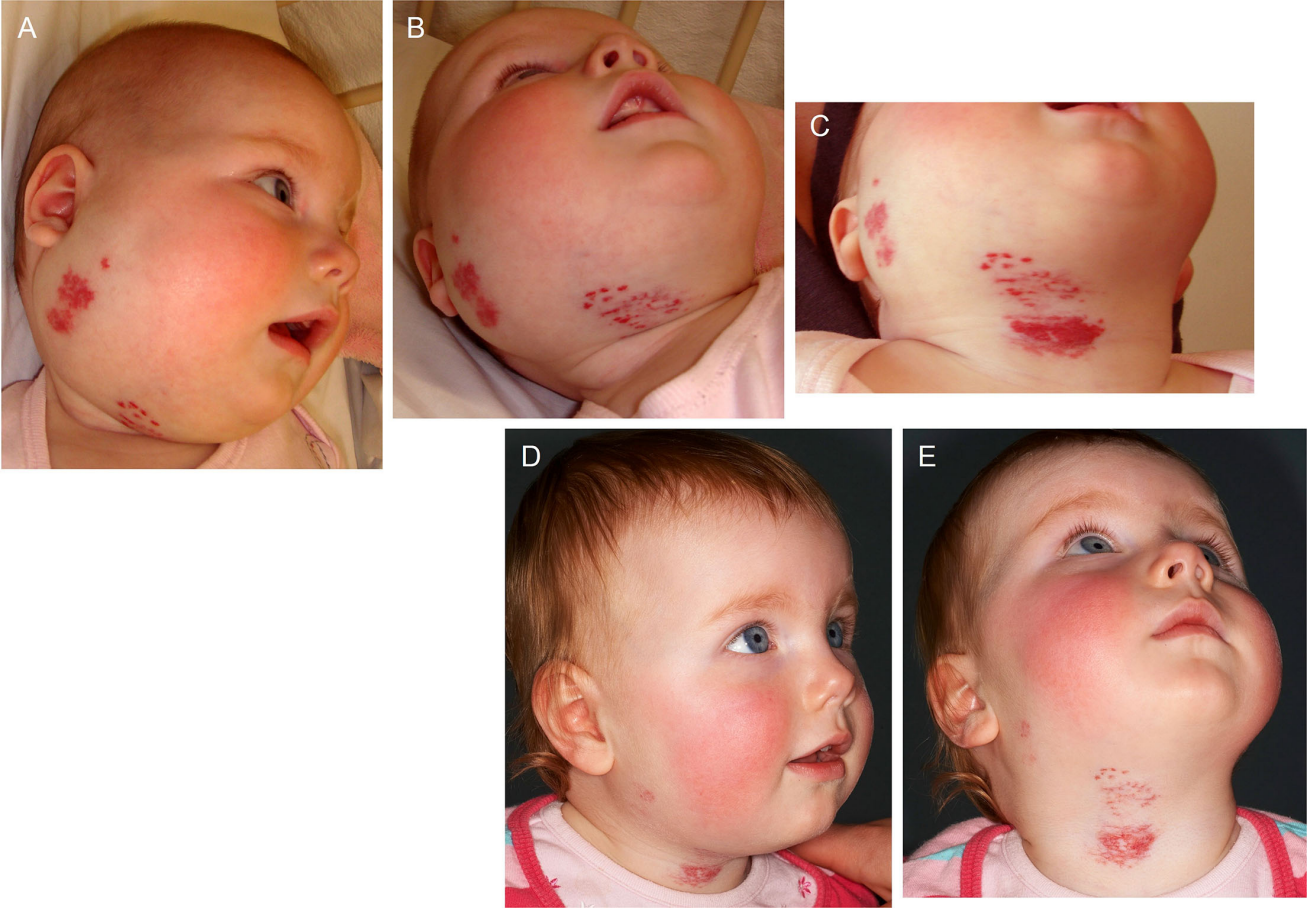
A 22-week-old girl with a 7 × 10 cm proliferating infantile hemangioma in the right cervicofacial area causing significant tissue distortion before **(A,B)**, 3 weeks **(C)** and 6 months **(D,E)** following administration of captopril at 1.5 mg/kg/day. *Reproduced with permission from the British Journal of Dermatology* (102).

The involvement of the RAS in IH is further supported by the demonstration of accelerated involution of problematic proliferating IH induced by the ACEI captopril (102) (Figure 4). Serum levels of renin, ACE and ATII are reduced in patients following surgical excision and propranolol treatment (87). Further, the level of serum ATII decreases following administration of the ACEI captopril, of IH (87).

Cathepsins B and D which constitute bypass loops of the RAS are expressed by the endothelium of the microvessels and cells in the interstitium (103), and cathepsin G (103) and chymase (104, 105) by mast cells within the interstitium, of IH. These bypass loops may enable continued production of ATII despite blockade with RASIs, and offer a potential explanation for the variable response of IH to β-blockers (85, 103).

Propranolol is administered as a racemic mixture of R- and S-propranolol. S-propranolol is an active enantiomer, and R-propranolol an inactive non-β-blocking enantiomer (106). Recent evidence shows that R-propranolol inhibits the growth of bEnd.3 hemangioma cells *in vivo*, independent of β-blockade (107). This is interesting, as R-propranolol inhibits the transcription factor SOX18. *SOX18* mutation causes hypotrichosis-lymphedema-telangiectasia and renal syndrome, which features lymphatic malformation. Propranolol as an effective long-term treatment for hypotrichosis-lymphedema-telangiectasia and renal syndrome, provides evidence it may cause accelerated involution of IH via a SOX18-dependent pathway, rather than just affecting the β-adrenergic receptors (106). These findings present an opportunity to investigate new therapeutic options for IH and other conditions using an enantiopure R(+) propranolol, instead of its conventional racemic mixture, allowing for a lower dosage and thus lower side effects of the treatment (106).

### Stem Cell Regulatory Pathways and the Renin-Angiotensin System in Other Vascular Anomalies

Components of the RAS are expressed by cells expressing stemness-associated markers in other types of VAs including pyogenic granuloma (77), VM (78), and LM (80).

**Figure 5:**
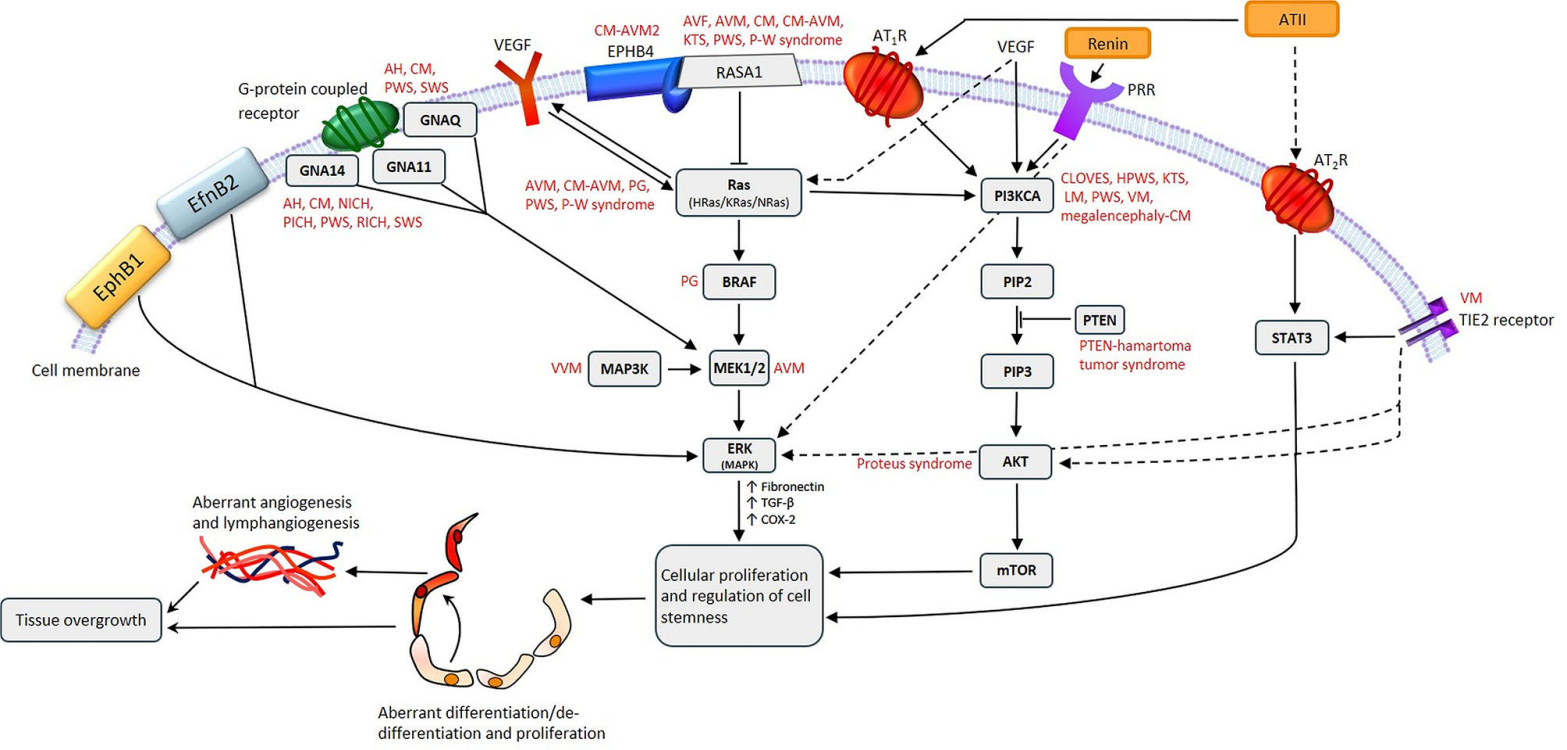
A proposed model for the role of gene mutations involving the Ras/BRAF/MEK/ERK and the PI3KCA/AKT/mTOR pathways by their interaction with different components of the renin-angiotensin system, leading to the induction and/or maintenance of cells that express stemness-associated markers in vascular anomalies. AH, anastomosing hemangioma; ATII, angiotensin II; AT_1_R, angiotensin II receptor 1; AT_2_R, angiotensin II receptor 2; PRR, pro-renin receptor; AVF, arteriovenous fistula; AVM, arterio-venous malformation; CM, capillary malformation; CM-AVM, capillary malformation—arterio-venous malformation; CM-AVM2, capillary malformation—arterio-venous malformation 2; CLOVES, congenital lipomatous overgrowth with vascular—epidermal and skeletal anomalies; HPWS, hypertrophic port-wine stain; P-W syndrome, Parkes-Weber syndrome; PWS, port-wine stain; KTS, Klippel-Trénaunay syndrome; LM, lymphatic malformation; NICH, non-involuting congenital hemangioma; PG, pyogenic granuloma; PICH, partially involuting congenital hemangioma; RICH, rapidly involuting congenital hemangioma; SWS, Sturge-Weber syndrome; VEGF, vascular endothelial growth factor; VM, venous malformation; VVM, verrucous venous malformation. For further information refer to “Cell Populations Expressing Stemness-Associated Markers in Vascular Anomalies” (1). *Reproduced with permission from Frontiers in Surgery* (1).

The RAS regulates stem cells (108) and interacts, at multiple levels, with the Ras/BRAF/MEK/ERK and PI3KCA/AKT/mTOR pathways which regulate cellular proliferation, differentiation, and stem cell maintenance (1) (Figure 5). Multiple gain-of-function gene mutations affecting these pathways that cause over-activity, have been identified in a number of VAs (1, 94, 109–111). The central role of the RAS in regulating hematopoietic cells, MSCs and angiogenesis, and the presence of components of the RAS (77, 78, 80, 102) and its bypass loops on ESC-like cells in VAs (103, 112–114), suggests possible therapeutic targeting of the ESC-like cells with RASIs in VAs (1).

Treatment of patients with CLOVES (a *PIK3CA*-related overgrowth syndrome) with the PIK3CA inhibitor BYL719P leads to improved symptoms with a decrease in the size of their VAs, and improved cardiovascular measures such as congestive heart failure, cardiac hemihypertrophy, and scoliosis (115). Vemurafenib restores normal blood flow of AVM in a BRAF-mutated zebrafish model (116). Low-dose AKT inhibitor miransertib (ARQ092) reduces the levels of phospho-AKT by approximately half in 83% of tissue samples of patients with Proteus syndrome, caused by gain-of-function mutations in AKT in the PI3KCA/AKT/mTOR pathway (117). Targeted therapies that inhibit MAPK2K1/ERK for these mutations have been investigated in many cancer types, including metastatic MM (118), advanced soft tissue sarcoma (119), and ganglioglioma (120). Targeting mutations in *MEK1/2 (MAP2K1)* have also been proposed for VAs. The interaction between the PI3CK/AKT/mTOR and RAF/MEK/ERK pathways via Ras may enable bypass of single step blockades by targeted therapies. In non-small cell lung cancer, *kras* mutations result in activation of the RAF/MEK/ERK pathway, where by inhibition of MEK with a MEK inhibitor is ineffective because of alternative activation of the PI3CK/AKT/mTOR pathway (121). Furthermore, resistance to MEK inhibitors may be worsened by activating mutations in *PI3CKA*, and by a mutation in *PTEN* (which inhibits PIK3CA) causing complete resistance (122). The potential of resistance to targeted therapies due to activation of alternative pathways, highlights the importance of continuing a broad search for safe and effective treatment strategies for VAs (1).

### The Efficacy of Sirolimus in Vascular Anomalies Further Supports the Role of Stem Cells in Vascular Anomalies

Sirolimus (rapamycin), a mammalian target of rapamycin (mTOR) inhibitor with anti-angiogenic, anti-lymphangiogenic, and anti-proliferative properties (123, 124), is traditionally used as an immunosuppressant in organ transplantation to prevent organ rejection (125). It is increasingly used for complex VAs (126) and *PIK3CA*-related overgrowth syndromes (127). mTOR is regulated by PI3K, which is involved in many cellular processes, including cellular proliferation, metabolism, autophagy, growth, as well as angiogenesis and lymphangiogenesis (126). Since its first use in an infant with kaposiform hemangioendothelioma refractory to conventional therapies (128), several studies have investigated its efficacy and safety profile for VAs. A systematic review by Freixo et al. (126) concludes that sirolimus improves outcomes for VAs, particularly vascular tumors associated with life-threatening coagulopathy, as well as LM and VM. In total 95.5% of patients with kaposiform hemangioendothelioma improve clinically, with correction of coagulopathy in 93% of patients. Size reduction is observed in 88.9% of patients with VM, and clinical improvement is experienced by 94.9% of patients with LM, following sirolimus treatment. However, no benefit of sirolimus has been demonstrated in patients with AVM. Of the 162 patients with vascular tumors and 211 with vascular malformations treated with sirolimus, multiple side effects were observed, including oral mucositis (31.9%), dyslipidemia (16.5%), leukopenia (12.3%), gastrointestinal symptoms (10.2%), and rash/eczema (8.2%) (126).

Sirolimus is involved in regulating cell stemness, which is relevant given the recent observation of cells expressing stemness-associated markers in many types of VAs (1). Short courses of sirolimus enhance somatic cell reprogramming, and longer-course treatment and mTOR-knockout reduces programming (129). Further, mTOR activation in human somatic cells with ectopic expression of OCT4, SOX2, KLF4 and c-MYC, increases the production of iPSCs (130). Interestingly, inhibiting mTOR induces a paused pluripotent state in mouse blastocysts, but maintains their phenotypic signature and pluripotency, suggesting that mTOR regulates developmental timing at the peri-implantation stage (131). The observed efficacy of sirolimus in VAs and the known role of mTOR in regulating stem cells, suggest a possible effect of mTOR inhibition on the primitive population within VAs (1). Sirolimus may alter or inhibit the stemness of these transcription factors within VAs, depending on the dose and duration of treatment (1).

In conclusion, VAs are a heterogeneous group of conditions that remain largely unsolved. The recent discovery of stem cells and the RAS in IH underscores the programmed biologic behavior and accelerated involution of proliferating IH induced by β-blockers and ACEIs, and provides a novel line of inquiry into other VAs. The efficacy of sirolimus in complex VAs, and the presence of ESC-like populations in multiple types of VAs points to ESC-like populations being a potential therapeutic target. Given the presence of components of the RAS and its bypass loops, it is interesting to speculate that these primitive populations may be targeted using RASIs.

## Cancer Stem Cells and the Renin-Angiotensin System in Cancer

### Cancer Stem Cells

**Figure 6:**
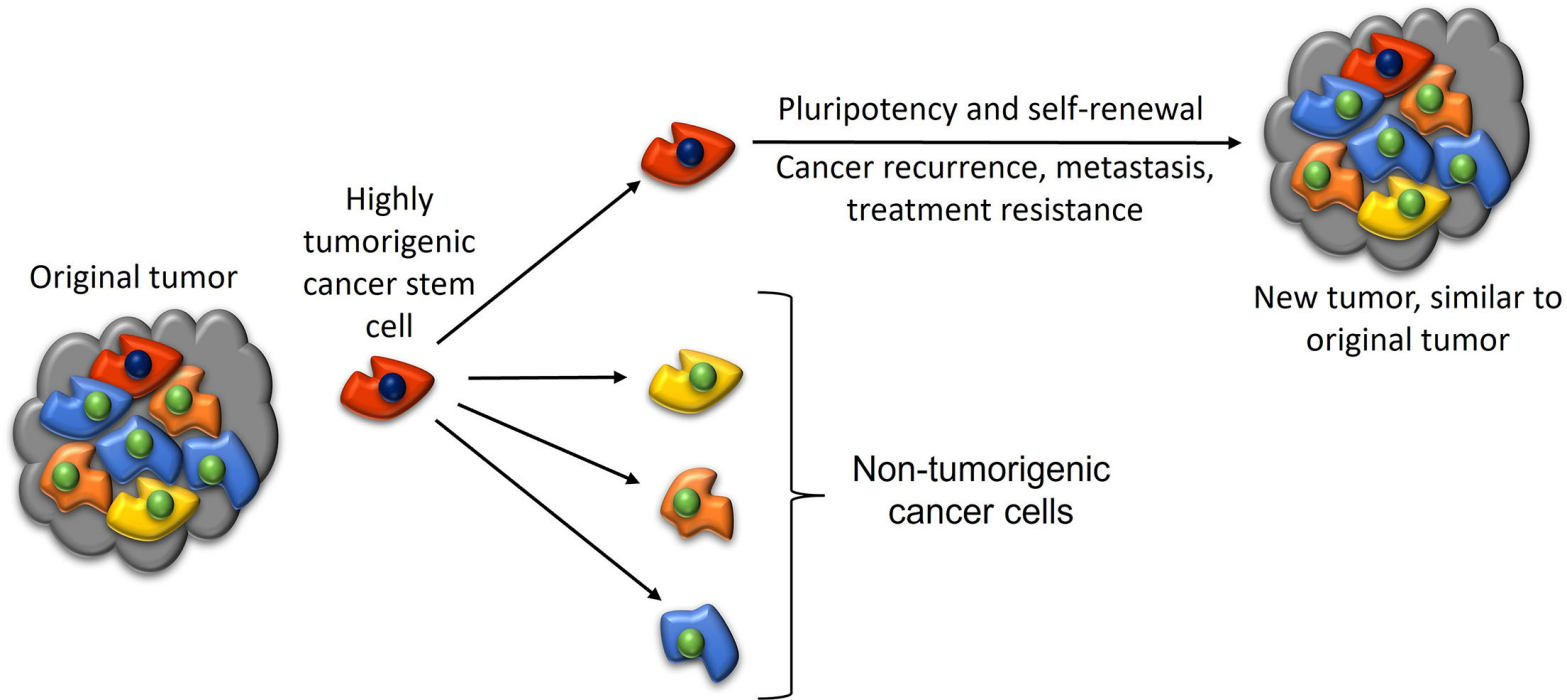
A diagram illustrating the cancer stem cell (CSC) concept of cancer. A highly tumorigenic CSC sits atop the tumor cellular hierarchy and divides asymmetrically to form non-tumorigenic cancer cells that form the bulk of the tumor, and identical CSCs that form new tumors that are similar to the original tumor. *Reproduced and modified with permission from the Atlas of Extreme Facial Cancer, Springer Nature, 2022* (6).

The CSC concept (Figure 6), also known as the hierarchical model of cancer, proposes that cancer progression is driven by CSCs (2). CSCs possess pluripotency and self-renewal properties, and are the proposed initiating cells and the driver of the tumor growth (2). CSCs are highly tumorigenic, divide symmetrically to form identical CSCs, and asymmetrically to form differentiated non-tumorigenic cancer cells that give rise to the bulk of the tumor (132). CSCs are attributed to tumor dormancy, metastasis (2), resistance to chemotherapy and radiotherapy (133) and immunotherapy (134), and recurrence (135).

CSCs have been demonstrated in many cancer types (2), including breast cancer (136), prostate cancer (137), glioblastoma (24), primary (22) and metastatic (26) colon adenocarcinoma, lung adenocarcinoma (23), oral cavity squamous cell carcinoma of different subsites (25, 28, 29), primary (30) and metastatic (12) HNcSCC, and metastatic MM to the regional nodes in the head and neck (31) and the brain (32).

Hypotheses on the origin of CSCs include de-differentiation of differentiated tissue somatic cells, or resident adult stem cells that become CSCs through genomic instability, an inflammatory tissue microenvironment, lateral gene transfer, and cell fusion (135).

Cancer metastasis is the main cause of cancer deaths (138). The “seed” and “soil” theory was first proposed in 1889, by which tumor cells (the seed) from the primary tumor travel to a distant organ (the soil) with a favorable environment for tumor cell colonization (139). Circulating tumor cells (CTCs) have been proposed to be responsible for metastasis (140). However, only a small proportion of CTCs possess metastatic potential, for example, 0.02% of melanoma cells injected into the portal circulation form macrometastases (141). CSCs are proposed to circulate in the circulatory system (142) and these may constitute the small fraction of CTCs that possess metastatic potential.

**Figure 7:**
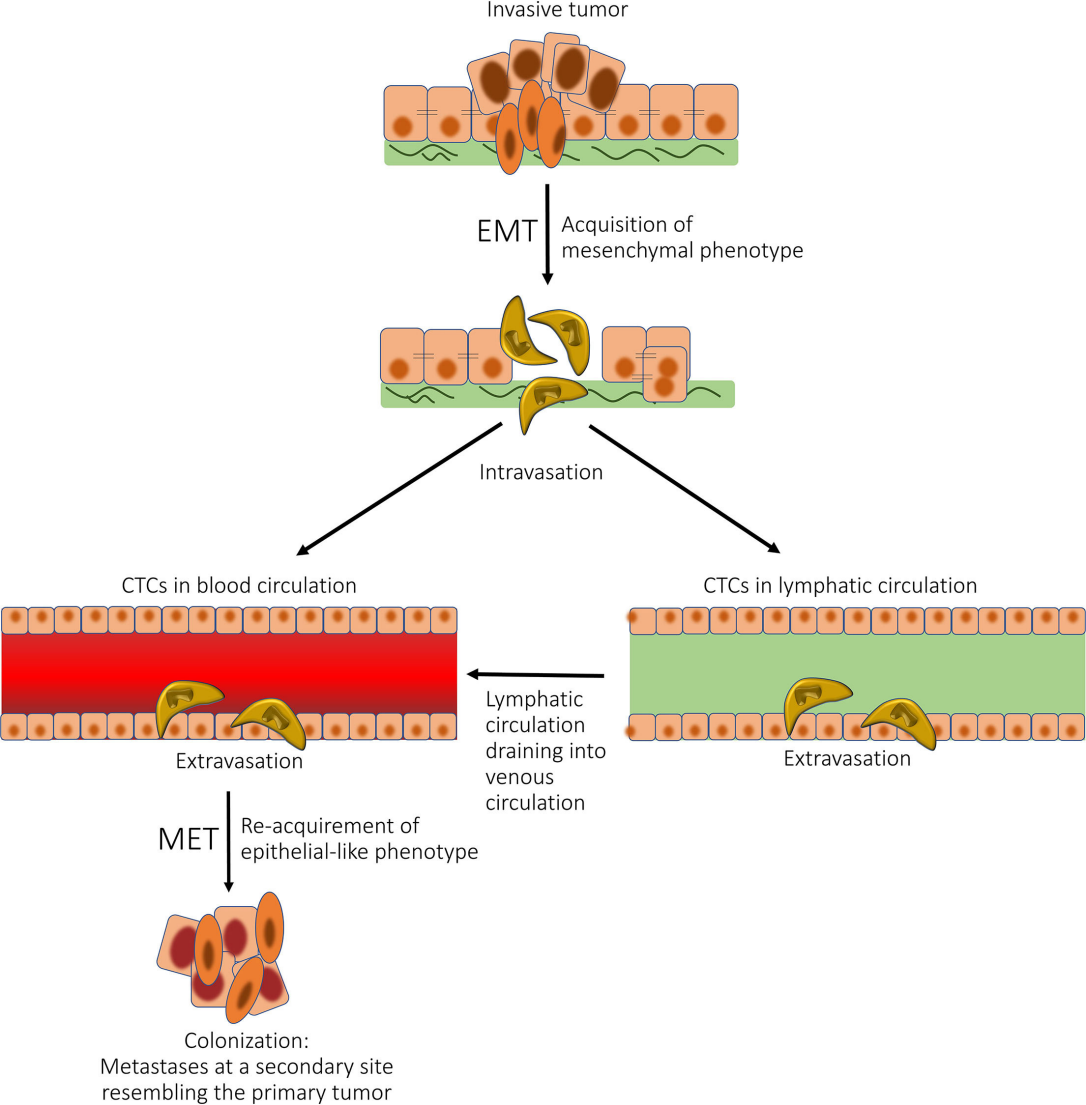
A diagram showing the role of epithelial-mesenchymal transition (EMT) and mesenchymal-epithelial transition (MET) in cancer metastasis. Tumor cells within a cancer undergo EMT to form mesenchymal-like cells, which undergo intravasation to enter the blood and/or lymphatic circulations as circulating tumor cells (CTCs). These CTCs then undergo MET and extravasate into distant tissue sites, where metastatic tumors may be established. *Reproduced with permission from the Atlas of Extreme Facial Cancer, Springer Nature, 2022* (6).

Cells within metastases phenotypically resemble CSCs (139). Tumor cells within a cancer undergo EMT to form mesenchymal-like cells that undergo intravasation to enter the blood and/or lymphatic circulations as CTCs. These CTCs that possess CSC characteristics undergo mesenchymal-to-epithelial transition (MET) and extravasate into distant tissue sites, where metastatic tumors may be established (Figure 7). MET is a fundamental process occurring during embryogenesis when the mesoderm becomes epithelial tissue in embryogenesis (143). CSCs may undergo EMT (144), which causes a phenotype conducive for tissue invasion and metastasis (145). It is interesting to speculate that a small proportion of CTCs have stemness properties, and a CSC-like phenotype, facilitating EMT and thus metastasis. EMT and MET play an important role in metastasis, which is intricately linked to epithelial plasticity (146). The role of CSCs in metastasis is supported by the demonstration that CD133^+^ and chemokine receptor CXCR4^+^ CSCs in pancreatic cancer have high metastatic potential, as do CD26^+^ CSCs in colon cancer (147, 148), and ALDH^+^ and CD44^+^/CD24^−^ CSCs in breast cancer (147).

### Modulation of the Renin-Angiotensin System and Cancer

Cutaneous SCC (cSCC) accounts for 15–25% of all skin cancers (149). The mainstay treatment of cSCC is surgical excision. About 2.6% of cases metastasize to the regional lymph nodes (150), with risk factors including tumor site, poor histologic differentiation, lymphovascular and/or perineural invasion (150), and tumor thickness and horizontal diameter (151, 152), and immunosuppression (153). The 5-year overall survival for metastatic HNcSCC is 48%, despite intensive treatment with surgery and adjuvant post-operative radiotherapy (154). These poor outcomes are attributed to the presence of CSCs (2, 12).

Populations of cells that express the stemness-associated markers OCT4, NANOG, SOX2, KLF4 and c-MYC, have been demonstrated in primary HNcSCC (12) and metastatic HNcSCC (30). Metastatic HNcSCC-derived primary cell lines are capable of forming tumorspheres, providing preliminary evidence of ESC-like cells within metastatic HNcSCC possessing stem cell function (12). It has been recently shown that *miR-22*, an miRNA important for follicle stem cell differentiation, promotes cancer development and metastasis by maintaining β-catenin signaling and hence CSC function (155). The CSC subpopulations in both primary HNcSCC (156) and metastatic HNcSCC (33) express components of the RAS.

MM constitutes 1% of all skin cancers but has the highest mortality among skin cancers with 6,850 deaths in the United States in 2020 (157). Primary MM has a five-year survival of over 90% (158), but this decreases to 43% in patients with regional metastasis (159). MM can develop in other organs, including the mucosal surface of the nasal cavity, paranasal sinuses, and the upper aerodigestive tract, gastrointestinal tract, vulva, and the central nervous system (158, 160, 161).

Multiple gain-of-function mutations in the mitogen-activated protein kinase (MAPK) pathway are often present in MM (162), which have been treated with targeted therapies (163–165). Advanced MM is treated with immunotherapeutic agents, such as the monoclonal antibody checkpoints cytotoxic T-lymphocyte antigen-4 (CTLA-4) with ipilimumab, and programmed death 1 (PD-1) with nivolumab and pembrolizumab. These agents significantly improve the life expectancy of patients with advanced MM and those at high risk of recurrence (166). However, primary or acquired resistance to anti-PD-1 or anti-PD-1/anti-CTLA-4 remains a clinical challenge, and encourages researchers to continue looking for non-immunotherapeutics that may be integrated with immunotherapy (166).

Propranolol is associated with improved cancer survival outcomes in patients with head and neck, esophageal, stomach, colon, and prostate cancer (167). A recent phase II clinical trial demonstrates that 1 week of pre-operative treatment with propranolol reduces biomarkers for metastasis in breast cancer (168). It has been hypothesized that RAS modulators such as propranolol exert their anti-cancer effects via β-adrenergic signaling which enhances tumor growth via β_2_-adrenergic receptors (169). A recent study shows that β_3_-adrenergic blockade with SR59230A reduces tumor growth and significantly attenuates the stemness markers CD44, NANOG, OCT3/4 and CD24, implicating β-adrenergic receptors in maintaining the stemness of CSCs within the TME (170). Propranolol, a non-selective β_1_- and β_2_-adrenergic receptor antagonist, inhibits renin secretion. As the RAS is a stem cell regulator, the anti-cancer effect of propranolol may be the result of its action on the local RAS within the TME. RAS modulators has been shown to reduce pluripotency and stem cell capability of colon adenocarcinoma-derived cells that possess stem cell function (171).

CSCs that express stemness-associated markers have been widely documented in MM (172, 173), including cells expressing OCT4, SOX2, KLF4, c-MYC in metastatic MM to the regional lymph nodes in the head and neck (31) and the brain (32). These cells are capable of forming tumorspheres *in vitro*, providing preliminary evidence of stemness properties (31). CSCs in metastatic MM to the regional nodes in the head and neck (171) and the brain (174) express components of the RAS. Ishikane et al. (173) demonstrate the important influence of the RAS on MM metastasis. Two weeks following injection of B16/F10 mouse melanoma cells that do not express AT_1_R, into C57BL/6 mice, the number of metastases is significantly greater in the group treated with ATII compared to the vehicle-treated group. The ARB valsartan, but not the calcium channel blocker amlodipine (which increases plasma renin levels), suppresses these effects of ATII. This data supports ATII in exacerbating hematogenous spread of MM by activating adhesion molecules in vascular endothelial cells. Renziehausen (175) demonstrates decreased expression and increased CpG island methylation of *AGTR1* within metastatic MM, compared to primary MM, implying that *AGTR1* acts as a tumor suppressor gene in MM. Furthermore, antagonizing AT_1_R with an ARB or shRNA-mediated knockdown in melanoma cells lines expressing *AGTR1*, allows the cell lines to proliferate in serum-free conditions. Interestingly, treatment with the AT_2_R antagonists PD123319 and EMA401 inhibits angiogenesis, melanoma cell growth, and increases the effect of BRAF and MEK inhibitors in cells with BRAF V600E mutations (175). The author proposes inhibition of AT_2_R as a potential therapeutic option for treating MM which expresses the RAS.

Another potential therapeutic benefit of RASIs is their enhancement of the effectiveness of existing cancer therapies. For example, RASIs increase the effects of anti-PD-1 therapy on MM. Investigation of the role of the RAS in the MM TME by Nakamura et al. (174) demonstrates that fibroblasts produce CC motif chemokine ligand 5 (CCL5) upon stimulation of the RAS, which is suppressed upon ARB administration in mice, and that ARB administration (1) decreases CCL5 blood concentration, (2) increases tumor-infiltrating T cells, (3) decreases regulatory T cells, and (4) increases tumor antigen-specific T-cell responses. Furthermore, ARB and anti-PD-1 co-administration significantly improves tumor growth inhibition, compared with monotherapy. This highlights the potential of RASIs as an adjuvant treatment to improve the therapeutic efficacy of anti-PD-1 antibodies and highlights the interaction between the RAS and the immune system in the TME. Further work is warranted to investigate whether RASIs improve the efficacy of other targeted therapies.

### Renin-Angiotensin System Inhibitors and Cancer Outcomes

In 1998, Lever et al. (176) first demonstrated that patients taking ACEIs and ARBs have a lower risk of several cancer types. Since then, there has been intensive investigation into the role of the RAS in cancer. The demonstration of components of the RAS in an increasing number of cancer types, and the emerging concept of a local tissue RAS and its associated non-classical roles, have led to the appreciation of the crucial role of the RAS in diseases including cancer, in addition to its classical endocrine effects (38).

Several observational epidemiological studies have shown a lower incidence of cancer and improved cancer outcomes in patients taking RASIs (3, 177) although some studies show no such association (41).

Propranolol increases the median progression-free survival and overall survival of patients with metastatic angiosarcoma by nine and 36 months, respectively (178). A systematic review and meta-analysis of 13 studies by Zhou et al. (179) demonstrates that administration of ACEIs or ARBs significantly improve survival outcomes in patients with gastrointestinal malignancies, with an improved overall survival (HR 0.79, 95%CI 0.70–0.89); *p* < 0.000), cancer-specific survival (HR 0.81, 95%CI 0.73–0.90; *p* < 0.000) and recurrence-free survival (HR 0.68, 95%CI 0.54–0.85; *p* = 0.001), but not progression-free survival (HR 0.88, 95%CI 0.73–1.07; *p* = 0.183) and disease-free survival (DFS; HR 0.50, 95%CI 0.11–2.39; *p* = 0.103). Another meta-analysis by Li et al. (180) demonstrates a significant improvement in overall survival (HR 0.80, 95%CI 0.69–0.92; *p* = 0.29) and progression-free survival (HR 0.79, 95%CI 0.66–0.94; *p* = 0.11) for those taking RASIs with chemotherapeutic agents, compared to those taking chemotherapy alone. A sub-group analysis demonstrates that platinum-based agents in conjunction with RASIs provides a significant benefit for pooled overall survival (HR = 0.56, 95%CI 0.38–0.82; *p* = 0.96) (180). A further meta-analysis of 55 studies by Sun et al. (177) also demonstrates significant improvement in overall survival (HR = 0.82, 95%CI 0.77–0.88; *p* = 0.001), disease-free survival (HR = 0.80, 95%CI 0.67–0.95; *p* = 0.01) and progression-free survival (HR = 0.74, 95%CI 0.66–0.84; *p* < 0.001) for those taking RASI.

Some randomized control trials (RCTs) show that RASIs have no effect on survival outcomes for cancer patients, and that some ARBs may increase cancer risk depending on the type of ARB. A decreased cancer incidence is associated with ARBs in observational studies (RR 0.91, 95%CI 0.84–.99; *p* = 0.022), however, there was no decrease in cancer incidence associated with ARBs in RCTs which was not statistically significant (RR 1.00, 95%CI 0.92–1.10; *p* = 0.964) (178). A recent meta-analysis of RCTs, which includes 68,402 patients with solid organ cancers from five trials, shows that ARBs are associated with a small increased risk of new cancer diagnosis. Patients randomly assigned to take ARBs have an increased risk of new cancer occurrence, compared with those in the control group (7.2 vs. 6.0%, RR 1.08, 95%CI 1.01–1.15, *p* = 0.016) (181). Furthermore, a protective effect of ACEIs on cancer development is not demonstrated (182), and another finds no association between ARB use and cancer risk (183). Lastly, ARB use has been associated with increased risk of lung cancer compared to patients in control groups (7.2 vs. 6.0%, risk ratio 1.08, 95% CI 1.01–1.15; *p* = 0.016) (181). The study design, the type of cancer studied, the duration of RAS agent administration, and the use of other medications are possible reasons for these conflicting results (184).

When analyzing epidemiological data on the effect of RASIs on cancer, ACE polymorphisms including the insertion/deletion (I/D), which influences the expression of ACE, should be considered. D/D carriers have higher ACE levels than I/I carriers, and the D/D polymorphism is associated with the extent of lymphatic metastases in gastric malignancy (179). D/D polymorphism has also been associated with worse prognosis in prostate cancer (185).

### Novel Therapeutic Targeting of the Renin-Angiotensin System in Cancer Treatment

**Figure 8:**
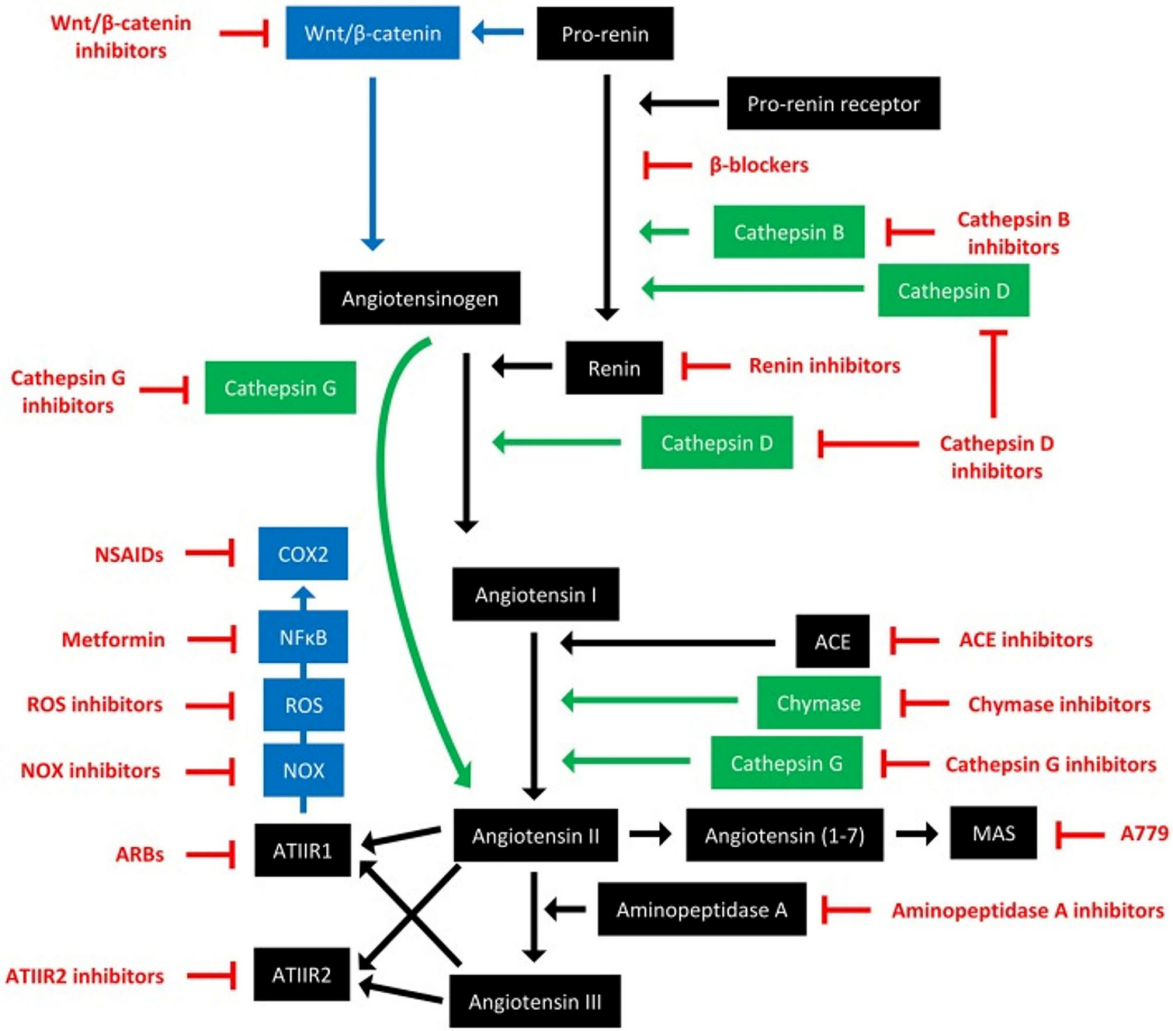
The renin-angiotensin system and its bypass loops and converging signaling pathways can be targeted at different points. The renin-angiotensin system (black) regulates blood pressure, stem cell differentiation, and tumor development. Bypass loops in the system involving cathepsins and chymase (green) provide redundancy, while convergent inflammatory and development signaling pathways (blue) have multiple roles and effects. Multiple points of the pathway can be targeted by specific inhibitors (red). ACE, angiotensin-converting enzyme; ARBs, AT_1_R blockers; ROS, reactive oxygen species; NSAIDS, non-steroidal anti-inflammatory drugs. For further information refer to ‘Therapeutic Targeting of Cancer Stem Cells via Modulation of the Renin-Angiotensin System’ (3). *Reproduced with permission from Frontiers in Oncology* (3).

The RAS, its bypass loops and converging signaling pathways can be inhibited at multiple points (Figure 8) (3). For example, renin can be directly inhibited with the renin inhibitor aliskerin, and its secretion blocked by β-blockers such as propranolol. ACE can be inhibited by ACEIs, and AT_1_R with ARBs. Cathepsins B, D, and G, and chymase, can be blocked by their respective inhibitors. Furthermore, signaling from AT_1_R impacts the NOX/ROS/NFkB/COX2 axis, which may be targeted with NOX inhibitors, ROS inhibitors, metformin, and non-steroidal anti-inflammatory drugs, respectively. Other converging signaling pathways of the RAS that can be targeted include Wnt/β-catenin signaling, which is influenced by pro-renin, which may be targeted by Wnt/β-catenin inhibitors. Lastly, aminopeptidase A can be targted with aminopeptidase A inhibitors, and MasR with its antagonist A779.

The RAS may affect the expression of stemess-associated markers in cancer (186) and CSCs either directly or indirectly via its influence on the TME directly and by its interaction with the immune system (5, 35). Targeting the RAS, its bypass loops and converging signaling pathways to achieve optimal blockade of the RAS to regulate CSCs has been proposed (3–5). This “hollistic” approach of blocking the RAS at multiple points simultaneously, forms the basis of a phase I clinical trial for recurrent glioblastoma (187) resulting in a trend toward increased survival by 5.3 months. The encouraging results of this trial warrant further clinical trials on this novel, well-tolerated and cost-effective potential therapeutic option for patients with glioblastoma, and potentially other cancer types.

The growing interest in combined drug treatment for cancer is highlighted by the treatment of recurrent glioblastoma using the CUSP9^*^ protocol, where nine repurposed drugs are used simultaneously (188) with three of the 10 patients remaining progression-free at 63, 54 and 52 months following tumor recurrence (Halatsch et al., personal communication, December 21, 2021). Aprepitant, artesunate, auranofin, captopril, celecoxib, disulfiram, itraconazole, ritonivir, and setraline which are used in the CUSP9^*^protocol, blocks one or more surival pathways used by glioblastoma cells, to potentially enhance the efficacy of temozolomide in the treatment of glioblastoma.

Taken together, the presence of CSCs – the proposed founding cells of cancer, and driver of metastasis, treatment resistance and recurrence, and the expression of componenets of the RAS and its bypass loops across multiple cancer types, raises the possibility of targeting CSCs with RASIs and inhibitors of RAS bypass loops.

## Embryonic Stem Cell-Like Cells, the Renin-Angiotensin System and Vitamin D in Fibroproliferative Conditions

Fibroproliferative conditions, characterized by excessive deposition of ECM, affect different organ systems including the heart, kidney and lung (189). KD and DD are fibroproliferative conditions commonly encountered by plastic surgeons for which current treatment remains empirical and unsatisfactory. Here we discuss the recent demonstration of ESC-like populations, their expression of components of the RAS and its bypass loops in KD and DD and its possible implications.

### Embryonic Stem Cell-Like Cells and the Renin-Angiotensin System in Keloid Disorder

KD is a fibroproliferative condition characterized by excessive dermal collagen deposition in response to skin injury, and/or inflammation (8). Keloid lesions (KLs) extend beyond the borders of the original wound, without spontaneous wound remodeling (190). KLs are rubbery plaque-like lesions that cause disfigurement, pruritis, tenderness, and functional problems such as joint contracture (191). KLs commonly occur on the earlobes, anterior chest, shoulders, and upper back, with increased sebaceous gland density and surface tension proposed as risk factors (8). Current treatments for KD include serial intralesional corticosteroid injections, and surgical excision with intra-operative steroid injections, cryotherapy, topical 5-fluorouracil, laser therapy, silicon occlusive dressing, and excision with post-operative radiotherapy (8).

**Figure 9:**
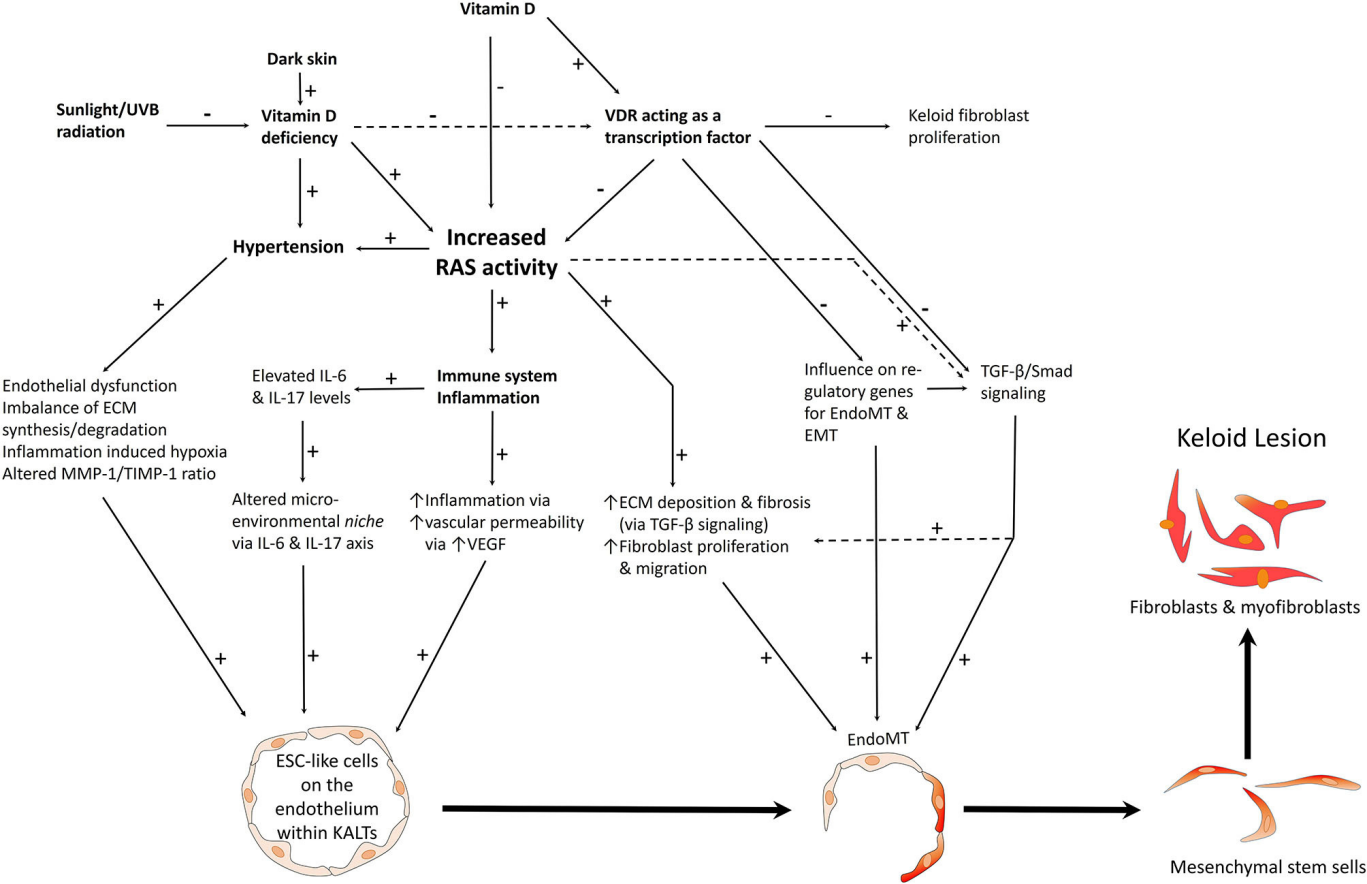
A proposed model of keloid disorder showing embryonic stem cell (ESC)-like cells within keloid-associated lymphoid tissues (KALTs), regulated by a microenvironmental niche with resultant proliferation and accumulation of fibroblasts and myofibroblasts in the keloid lesion (KL) via a mesenchymal stem cell intermediate through an endothelial-to-mesenchymal transition (endoMT). The renin-angiotensin system (RAS) plays a central role in the microenvironmental niche with complex interactions with the immune system/inflammation, vitamin D, vitamin D deficiency (VDD), vitamin D receptor (VDR), and hypertension. VDD which is caused by reduced sunlight/UVB radiation, and leads to increased RAS activity and the resultant hypertension. VDD also directly leads to hypertension. Increased RAS activity also activates the immune system. The complex interactions between these elements lead to activation of various pro-fibroproliferative signaling pathways leading to generation of fibroblasts and myofibroblasts. Hypertension has a direct pro-fibroproliferative effect and contributes to the conducive microenvironment for the ESC-like cells within the KALTs. VDD increases RAS activity, with activation of the immune system/inflammation leading to an altered microenvironmental niche via the IL-6 and IL-17 axis. This increased RAS activity activates TGF-β/Smad signaling to promote EndoMT. Binding of vitamin D to VDR results in a genomic effect which counteracts the pro-fibroproliferative signaling pathways. VDR transcriptional activity inhibits keloid fibroblast proliferation. VDR transcriptional activity also inhibits the pro-fibroproliferative TGF-β/Smad signaling pathway, down-regulates genes for EndoMT, and so may influence the formation of fibroblasts and myofibroblasts within KLs. ECM, extracellular matrix; TGF-β, transforming growth factor-β; MMP-1, matrix metalloproteinase-1; TIMP-1, tissue inhibitor of metalloproteinase-1; IL, interleukin; UVB, ultraviolet B; VEGF, vascular endothelial growth factor. “+” signifies a positive effect; “–” signifies a negative effect. For further information refer to “The Role of the Renin-Angiotensin System and Vitamin D in Keloid Disorder — A Review” (8). *Reproduced with permission from Frontiers in Surgery* (8).

ESC-like cells have been demonstrated in keloid-associated lymphoid tissues (KALTs) (20) consisting of aggregates of lymphoid cells expressing CD20, located in the reticular dermis, beneath the epidermis of KLs (192). This primitive population expresses the stemness-associated markers OCT4, SOX2, and pSTAT3 which are localized to the endothelium and perivascular cells of the KALTs (20). mRNA of the stemness-associated marker Rex-1 is also present in KLs, and SSEA-4 has been demonstrated in the reticular dermis of KLs (193) (Figure 9).

PRR, ACE, AT_1_R and AT_2_R are expressed on the ESC-like cells on the endothelium, with PRR also present on the pericyte layer, of the microvessels within the KALTs (194). Vitamin D receptor (VDR) is also expressed on the endothelium of the microvessels and perivascular cells within the KALTs (19). These ESC-like cells in KLs also express cathepsins B, D and G – proteases that act as bypass loops of the RAS (192).

Sustained activation of the RAS results in hypertension and organ fibrosis (195) via AT_1_R signaling and an ATII-mediated increase in TGF-β (196). AT_1_R signaling increases cellular proliferation, oxidative stress, inflammation, and creates a pro-fibroproliferative environment via inflammatory cell recruitment, and with other microenvironmental factors, ultimately resulting in excessive ECM deposition (197).

Dark-skinned individuals are predisposed to vitamin D deficiency (VDD) (198) which increases RAS activity via the genomic actions of VDR (199). The RAS alters the microenvironmental niche within KLs by influencing the immune system, the IL-6 and IL-17 axis, and by increasing vascular permeability and inflammation (8). Acting as a transcription factor, VDR signaling contributes to the formation of keloid fibroblasts and TGF-β/smad signaling, and may alter the expression of genes regulating endothelial-to-mesenchymal transition (endoMT) and EMT (8, 9, 200). Elevated RAS activity also increases ECM deposition and fibrosis via increased TGF-β signaling and increased fibroblast proliferation and migration (201). This complex network of interactions results in the accumulation of aberrant fibroblasts and myofibroblasts, via a MSC intermediate, from endoMT (8) (Figure 9). ATII mediates endoMT and RAS inhibition, with the ARB irbesartan preventing glucose-mediated increase in α-SMA in endoMT (202).

Dark-skinned individuals are predisposed to KD (203), VDD (204) and hypertension (205). The observation that VDD and hypertension are associated with elevated activity of the RAS (195, 199), and the expression of components of RAS by ESC-like cells with in KALTs, point to the central role of the RAS in the pathogenesis of KD (8). The RAS may influence the microenvironmental niche, through its interactions with VDR-mediated genomic mechanisms and the immune system, to regulate the ESC-like population within the KALTs, leading to the proliferation and accumulation of aberrant myofibroblasts and fibroblasts (Figure 9).

### Vitamin D Metabolism and Actions

Vitamin D (VD) in the body exists in two forms: vitamin D_2_ (VD_2_, ergocalciferol) and vitamin D_3_ (VD_3_, cholecalciferol). VD_2_ is gained from irradiation of plant sterol, ergosterol, and from dietary sources (206). Key determinants of VD_3_ production include ultraviolent B intensity, which depends on latitude (204), and skin pigmentation which is determined by melanin pigment (207). Endogenous and exogenous VD are inactive and require two sequential hydroxylations to produce active VD (1,25-hydyoxyvitamin D_3_) (208). The first hydroxylation step occurs in the liver, where 25-hydroxylation generates 25-OH-cholecalciferol (calcidiol). This then binds to VD binding protein and is released into the circulation to form the main circulating state of VD, and is therefore used as a marker of VD status (208). The second step is 1α-hydroxylation by 1α-hydroxylase, which is encoded by *CYP27B1*, and may be classified as renal-hydroxylation, occurring in the kidney, or extra-renal hydroxylation, occurring in other tissues. This converts 25-hydroxycholecalciferol (calcifediol) into the active form, 1,25-hydyoxyvitamin D_3_ (calcitriol). This is largely responsible for the calcemic effects of VD. Extra-renal 1α-hydroxylation occurs in multiple organs and cell types – including kidney, brain, immune cells, lung, placenta and prostate (206). VD_2_ and VD_3_ will both be referred to as VD, due to their comparable biologic activity upon binding to VDR (209).

VD has its effect via VDR through both genomic and non-genomic actions (209). Its non-genomic actions include regulation of intestinal calcium and phosphorus absorption (210). For its genomic actions, VDR can change the expression of genes involved in cellular proliferation, stemness, and immunomodulation (206, 209). 1,25-hydyoxyvitamin D_3_ binds to VDR, which then interacts with the retinoid X receptor which forms a heterodimer that binds to VD responsive elements near genes regulated by VD (208). This causes ligand-dependent induction or repression of gene transcription, alongside other recruited repressors and activators. The sheer quantity of target genes accounts for the broad pleiotropic actions of VDR (211). Given the presence of VDR in KLs, it is interesting to speculate its potential genomic actions on aberrant fibroblasts and myofibroblasts within KD, upon binding of VD (Figure 9).

### Extra-Renal Hydroxylation of 25-Hydroxyycholecalciferol in Keloid Disorder

Various extra-renal tissues express 1α-hydroxylase which produces 1,25-hydyoxyvitamin D_3_ which acts locally in a tissue microenvironment via VDR. Notably, T and B lymphocytes produce the active form of VD. As mentioned, OCT4^+^ ESC-like cells within the KALTs of KLs express the VDR (19). It is interesting to speculate that active VD binds to VDR to exert genomic mechanisms on the ESC-like cells, and other cells expressing VDR in the tissue microenvironment to influence the local conditions to affect cell proliferation, stemness, and the chronic immune response seen in KD (8) (Figure 9). It is known that VD acts locally, regulating cell growth and proliferation within tissues such as the prostate and the placenta. Unlike renal 1α-hydroxylase, extra-renal 1α-hydroxylase is stimulated by cytokines, not parathyroid hormone (212).

### Embryonic Stem Cell-Like Cells and the Renin-Angiotensin System in Dupuytren's Disease

DD affects the palmar fascia of the hand (213) with a predilection for those of Northern European descent, affecting almost 30% of the Norwegian population over 60 years (214). Fasciectomy is the first-line treatment for DD, which involves surgical removal of the fibrous cords and nodules, with a recurrence rate of up to 66% (215). More recently, non-operative treatment with *Clostridium histolyticum* collagenase injection has been used (216). The long-term outcomes and complications remain unknown, with repeated use being associated with neuromuscular and coagulation disorders (217, 218).

**Figure 10:**
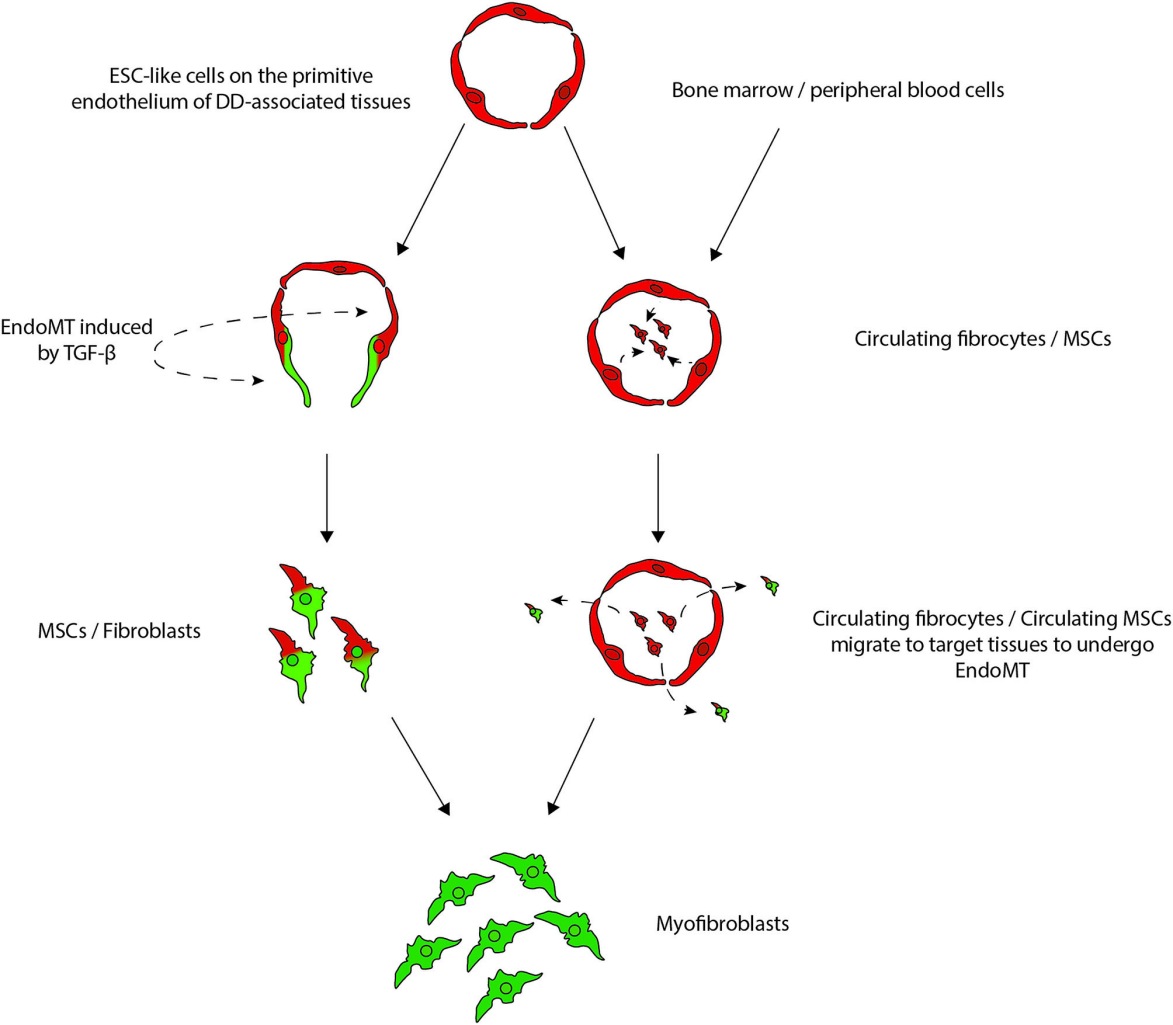
A proposed model demonstrating the potential sources of stem cells that give rise to the myofibroblasts observed in Dupuytren's disease (DD): (1) DD tissue-associated embryonic stem cell (ESC)-like cells localized to the endothelium of the microvessels that undergo endothelial-to-mesenchymal transition (EndoMT) induced by cytokines such as TGF-β_1_, giving rise to myofibroblasts through a mesenchymal stem cell (MSC) intermediate; or (2) circulating fibrocytes and circulating MSCs that originate from the bone marrow or the peripheral blood cells or from the primitive endothelium of the microvessels in DD-associated tissue, and migrate to DD sites and differentiate into myofibroblasts, via an EndoMT. *Reproduced with permission from Plastic and Reconstructive Surgery – Global Open* (7).

An ESC-like population expressing OCT4, NANOG, SOX2, pSTAT3 and SALL4 has been demonstrated on the endothelium of the microvessels in the cords and nodules of DD (21), in the location of a reported MSC population that expresses CD13 and CD29 (213). This MSC population may act as a reservoir for the aberrant myofibroblast population in DD (213) (Figure 10). Interestingly, dermatofasciectomy which involves excision of the overlying skin and surrounding tissues with full-thickness skin grafting, lowers the recurrence rate to 11.6% (215). This suggests the tissue surrounding the DD cords and nodules is involved in the disease process, potentially acting as a reservoir of the progenitor cells that give rise to aberrant myofibroblasts giving rise to DD (21, 217). Resident cells that differentiate into aberrant myofibroblasts in fibroproliferative conditions, have also been reported in other organs such as the liver and skin (8, 217).

The ESC-like population on the endothelium of the microvessels surrounding cords and nodules of DD expresses components of the RAS: PRR, ACE, AT_1_R, AT_2_R, with ACE and PRR also expressed on the pericytes (36). Stephen et al. (49) report AT_2_R being more abundant than AT_1_R in DD (218), which is interesting given the largely unknown role of AT_2_R, which is expressed abundantly in fetal tissue compared to adult tissues. This suggests a potential therapeutic role of AT_2_R antagonists and ACEIs for DD (219). The proteases cathepsins B, D and G, which constitute bypass loops of the RAS are expressed on the cords and nodules in DD with cathepsins B and D being expressed by the ESC-like cells on the endothelium of the microvessels (220). As the RAS is an important stem cell regulator (108), the expression of components of the RAS and cathepsin D on the ESC-like cells within DD, suggests this primitive population could be a potential therapeutic target by modulation of the RAS.

The ESC-like population on the microvessels has been proposed to be the source of the aberrant myofibroblasts in DD, via an MSC intermediate. This may occur by the primitive population acquiring an ESC-like phenotype, possibly under the influence of microenvironmental factors including the RAS, with a subsequent phenotypic shift giving rise to MSCs via endoMT, as proposed for KD (8).

Circulating CD34^+^ fibrocytes and MSCs from the bone marrow have also been proposed as another source of aberrant myofibroblasts (7) (Figure 9). Fibrocytes are fibroblast progenitor cells that originate from the bone marrow that migrate to distant sites where they participate in inflammation and fibrosis (219, 221). Circulating fibrocytes that exhibit a connective tissue myeloid cell phenotype are implicated in the pathogenesis of other fibroproliferative conditions. As progenitor cells, fibrocytes can give rise to cells of mesenchymal lineage, including myofibroblasts, under cues from the tissue microenvironment. Under the influence of growth factors, such as TGF-β and endothelin, fibrocytes gain the expression of α-smooth-muscle actin to turn into myofibroblast-like cells. The RAS cooperates with TGF-β to induce fibrosis via the AT_1_R receptor in many tissue types (201).

### Clinical Use of RAS Modulators in Keloid Disorder and Dupuytren's Disease

There is a paucity of pre-clinical studies investigating the functional role of the RAS in DD with conflicting results, and there are no clinical studies investigating the effect of RASIs on DD (36, 219, 222, 223). There are a number pre-clinical and small clinical studies investigating the effect of RASIs in KD with mixed results (8). A pilot placebo-controlled trial investigating the effect of 5% topical losartan on 37 adults shows that the Vancouver scar scale scores reduce significantly in the losartan group with both KD and hypertrophic scars, with markedly reduced vascularity and improved pliability (222). Topical ACEI has been reported to improve KL in a case report of a post-burn patient (223), and in a study of two patients (224). Further investigations of the role of the RAS in KD and DD including functional studies and larger clinical trials are warranted to elucidate the effectiveness of RAS modulators in these fibroproliferative conditions.

## Targeting the Renin-Angiotensin System in Vascular Anomalies, Cancer, and Fibroproliferative Conditions: Future Directions

There is increasing evidence implicating ESC-like cells and the RAS in VAs, cancer, and fibroproliferative conditions.

Gain-of-function mutations affecting the Ras/BRAF/MEK/ERK1/2 and PI3KCA/AKT/mTOR pathways have been identified in many types of cancer and VAs. These pathways regulate cellular proliferation and stemness, and interact with the RAS at multiple points (1). The RAS modulates cellular invasion, migration, proliferation, and angiogenesis. It is an important stem cell regulator. It also indirectly influences ESC-like cells via its direct influence on the tissue microenvironment and indirectly by its interaction with the immune system (5).

The discovery of ESC-like cells that express components of the RAS in IH underscores the recent paradigm shift in the understanding of the programmed biologic behavior and accelerated involution of this vascular tumor, induced by β-blockers and ACEIs. The findings of SOX18 inhibition by R-propranolol suggests the possibility of targeting ESC-like cells in IH without β-adrenergic blockade, and its associated side effects. Further research is required to elucidate how SOX18 impacts the ESC-like cells in IH, and other VAs, cancer and fibroproliferative conditions. Using enantiopure R(+) propranolol instead of its typical racemic mixture may enable a lower dosage, and thus fewer side effects of the treatment (106).

The efficacy of the mTOR inhibitor sirolimus, and targeted therapies that block the Ras/BRAF/MEK/ERK1/2 and PI3KCA/AKT/mTOR pathways in cancer and complex VAs, further supports a role of ESC-like cells in the pathogenesis of these conditions. Inhibition of these pathways leading to modification of the behavior of the ESC-like cells may account for the clinical efficacy of these therapies (1).

Targeting the RAS may directly affect ESC-like cells. It is interesting to speculate this may be in part due to the alteration of over-activity in the Ras/BRAF/MEK/ERK and PI3KCA/AKT/mTOR pathways, which are commonly affected by gain-of-function mutations in various types of cancer and VAs. Further research is required to understand the precise effect of the RAS on the ESC-like cells in these conditions, and the activity of the Ras/BRAF/MEK/ERK and PI3KCA/AKT/mTOR pathways. The hypothesis that the observed efficacy of the mTOR inhibitor sirolimus in VAs may be attributed to its effect on the stemness and behavior of ESC-like cells (1) also warrants further investigation.

The growing health impacts (225) and economic cost associated with escalating cost of novel cancer treatments, has given the impetus to search for an affordable, effective, safe and well-tolerated therapy through drug repurposing (226). *In vitro* studies have shown that RAS inhibition suppresses the hallmarks of cancer in different experimental models (225). There is a body of evidence demonstrating the role of the RAS in cancer, and the beneficial effect of RASIs in cancer outcomes, and in enhancing the effects of traditional cancer therapies (68).

The RAS, its bypass loops and converging pathways can be inhibited at multiple steps with existing medications (Figure 8). This offers an exciting opportunity in developing novel therapeutic approaches to achieve optimal modulation of the crucial and complex system by further investigations. This novel approach of blocking the RAS at multiple points simultaneously forms the basis of a phase I clinical trial for glioblastoma (187). The encouraging results of this trial warrant further clinical trials on this potential novel, well-tolerated and cost-effective therapeutic option for patients with glioblastoma, and potentially other cancer types.

Further investigations into the role of ESC-like cells and the RAS in VAs, cancer, and fibroproliferative conditions, may lead to novel therapeutic approaches to the treatment of these hitherto unsolved problems in plastic surgery.

## Author Contributions

EK drafted the manuscript. ST critically revised the manuscript. Both authors approved the final version of the manuscript.

## Conflict of Interest

ST is an inventor of the patents Cancer Diagnosis and Therapy (PCT/NZ2015/050108, AUS/2012302419, JAP/2017528398, and US/0281472), Cancer Therapeutic (PCT/NZ2018/050006), Novel Pharmaceutical Compositions for Cancer Therapy (PCT/NZ2019/050087), Treatment of fibrotic conditions (PCT/NZ2016/050187), Treatment of vascular anomalies (PCT/NZ2017/050032), and Methods and compositions for the treatment of hemangioma (PCT/NZ2021/050012). The remaining author declares that the research was conducted in the absence of any commercial or financial relationships that could be construed as a potential conflict of interest.

## Publisher's Note

All claims expressed in this article are solely those of the authors and do not necessarily represent those of their affiliated organizations, or those of the publisher, the editors and the reviewers. Any product that may be evaluated in this article, or claim that may be made by its manufacturer, is not guaranteed or endorsed by the publisher.

## Abbreviations

ACE: angiotensin-converting enzyme
ACEIs: angiotensin-converting enzyme inhibitors
AGT: angiotensinogen
ATI: angiotensin I
ATII: angiotensin II
ATIII: angiotensin III
ATIV: angiotensin IV
ARBs: angiotensin receptor blockers
AT_1_R: angiotensin II receptor 1
AT_2_R: angiotensin II receptor 2
AVM: arterio-venous malformation
BM: bone marrow
CCL5: chemokine (CC motif) ligand 5
CD20: cluster of differentiation 20
CD24: cluster of differentiation 24
CD29: cluster of differentiation 29
CD44: cluster of differentiation 44
CD133: cluster of differentiation 133
CI: confidence interval
CSCs: cancer stem cells
CTCs: circulating tumor cells
CTLA-4: cytotoxic T-lymphocyte antigen-4
DD: Dupuytren's disease
ECM: extra-cellular matrix
EMT: epithelial-to-mesenchymal transition
endoMT: endothelial-to-mesenchymal transition
ESC: embryonic stem cell
HNcSCC: head and neck cutaneous squamous cell carcinoma
IH: infantile hemangioma
iPSCs: induced pluripotent stem cells
IL: interleukin
KALTs: keloid-associated lymphoid tissues
KD: keloid disorder
KLs: keloid lesions
KLF4: Krüppel-like factor 4
LM: lymphatic malformation
MM: malignant melanoma
MAPK: mitogen-activated protein kinase
MasRs: Mas receptors
MrgDs: Mas-related-G protein coupled receptors
MSCs: mesenchymal stem cells
mTOR: mammalian target of rapamycin
OCT4: octamer-binding transcription factor 4
PD-1: programmed death 1
PTEN: phosphatase and tensin homolog
PRR: pro-renin receptor
PWS: port-wine stain
RAS: renin-angiotensin system
RASIs: renin-angiotensin system inhibitors
RCTs: randomized controlled trials
SALL4: Sal-like protein 4
SOX2: sex determining region Y-box 2
SOX18: sex determining region Y-box 18
SSEA-4: stage-specific antigen-4
STAT-3: signal transducer and activator of transcription-3
TGF-β: transforming growth factor-β
TME: tumor microenvironmental
VAs: vascular anomalies
VD: vitamin D
VDD: vitamin D deficiency
VDR: vitamin D receptor
VM: venous malformation.

## References

[1] Kilmister EJ Hansen L Davis PF Hall SRR Tan ST. Cell populations expressing stemness- associated markers in vascular anomalies. Front Surg. (2021) 7:1–18. 10.3389/fsurg.2020.610758 33634164 PMC7900499

[2] Batlle E Clevers H. Cancer stem cells revisited. Nat Med. (2017) 23:1124–34. 10.1038/nm.4409 28985214

[3] Roth IM Wickremesekera AC Wickremesekera SK Davis PF Tan ST. Therapeutic targeting of cancer stem cells via modulation of the renin-angiotensin system. Front Oncol. (2019) 9:745. 10.3389/fonc.2019.00745 31440473 PMC6694711

[4] Tan DC Roth IM Wickremesekera AC Davis PF Kaye AH Mantamadiotis T. Therapeutic targeting of cancer stem cells in human glioblastoma by manipulating the renin-angiotensin system. Cells. (2019) 8:1364. 10.3390/cells8111364 31683669 PMC6912312

[5] Kilmister EJ Tan ST. The role of the renin–angiotensin system in the cancer stem cell niche. J Histochem Cytochem. (2021) 69:835–47. 10.1369/00221554211026295 34165363 PMC8647629

[6] Kilmister EJ Tan ST. Cancer stem cells in head and neck cancers. In: Burton I. editor Atlas of Extreme Facial Cancer Springer Nature AG. (2022).

[7] Tan K Withers AHJ Tan ST Itinteang T. The role of stem cells in dupuytren's disease: a review. Plast Reconstr Surg - Glob Open. (2018) 6. 10.1097/GOX.0000000000001777 29922559 PMC5999435

[8] Kilmister EJ Paterson C Brasch HD Davis PF. The role of the renin-angiotensin system and vitamin D in keloid disorder-a review. Front Surg. (2019) 6:67. 10.3389/fsurg.2019.00067 32039229 PMC6988818

[9] Lim KH Itinteang T Davis PF Tan ST. Stem cells in keloid lesions: a review. Plast Reconstr Surg - Glob Open. (2019) 7:e2228. 10.1097/GOX.0000000000002228 31333955 PMC6571348

[10] Evans MJ Kaufman MH. Establishment in culture of pluripotential cells from mouse embryos. Nature. (1981) 292:154–6. 10.1038/292154a0 7242681

[11] Takahashi K Tanabe K Ohnuki M Narita M Ichisaka T Tomoda K. Induction of pluripotent stem cells from adult human fibroblasts by defined factors. Cell. (2007) 131:861–72. 10.1016/j.cell.2007.11.019 18035408

[12] Kilmister EJ Patel J Van Schaijik B Bockett N Brasch HD Paterson E. Cancer stem cell subpopulations are present within metastatic head and neck cutaneous squamous cell carcinoma. Front Oncol. (2020) 10:1091. 10.3389/fonc.2020.01091 32850316 PMC7406827

[13] Itinteang T Withers AHJ Davis PF Tan ST. Biology of infantile hemangioma. Front Surg. (2014) 1:38. 10.3389/fsurg.2014.00038 25593962 PMC4286974

[14] Blackwell MG Itinteang T Chibnall AM Davis PF Tan ST. Expression of embryonic stem cell markers in pyogenic granuloma. J Cutan Pathol. (2016) 43:1096–101. 10.1111/cup.12786 27509392

[15] Luke Krishnan CS Brasch HD Patel J Bockett N Paterson E. Stemness-associated markers are expressed in extracranial arteriovenous malformation. Front Surg. (2021) 8:31. 10.3389/fsurg.2021.621089 33816543 PMC8017302

[16] Tan EMS Siljee SD Brasch HD Enriquez S Tan ST Itinteang T. Embryonic stem cell-like subpopulations in venous malformation. Front Med. (2017) 4:162. 10.3389/fmed.2017.00162 29046873 PMC5632722

[17] Laing EL Brasch HD Steel R Jia J Itinteang T Tan ST. Verrucous hemangioma expresses primitive markers. J Cutan Pathol. (2013) 40:391–6. 10.1111/cup.12078 23379586

[18] Williams J Brasch HD Bockett N Patel J Paterson E Davis PF. Embryonic stem cell-like population in hypertrophic port-wine stain. J Vasc Anomalies. (2021) 2:e006. 10.1097/JOVA.0000000000000006

[19] Kilmister EJ Lim KH Itinteang T Van Schaijik B Brasch HD Davis PF. Keloid-associated lymphoid tissues in keloid lesions express vitamin D receptor. Int J Clin Exp Pathol. (2019) 12:3027–31. 31934141 PMC6949726

[20] Grant C Chudakova DA Itinteang T Chibnall AM Brasch HD Davis PF. Expression of embryonic stem cell markers in keloid-associated lymphoid tissue. J Clin Pathol. (2016) 69:643–6. 10.1136/jclinpath-2015-203483 27030305

[21] Koh SP On N Brasch HD Chibnall AM Armstrong JR Davis PF Tan ST Itinteang T. Embryonic stem cell-like population in Dupuytren's disease. Plast Reconstr Surg - Glob Open. (2016) 4:e1064. 10.1097/GOX.0000000000001064 27975007 PMC5142473

[22] Munro MJ Wickremesekera SK Peng L Marsh RW Itinteang T Tan ST. Cancer stem cell subpopulations in primary colon adenocarcinoma. PLoS ONE. (2019) 14:e0221963. 10.1371/journal.pone.0221963 31491003 PMC6730900

[23] Paterson C Kilmister EJ Brasch HD Bockett N Patel J Paterson E. Cell populations expressing stemness-associated markers in lung adenocarcinoma. Life. (2021) 11:1106. 10.3390/life11101106 34685477 PMC8541371

[24] Bradshaw A Wickremsekera A Tan ST Peng L Davis PF Itinteang T. Cancer stem cell hierarchy in glioblastoma multiforme. Front Surg. (2016) 3:1. 10.3389/fsurg.2016.00021 27148537 PMC4831983

[25] Baillie R Itinteang T Yu HH Brasch HD Davis PF Tan ST. Cancer stem cells in moderately differentiated oral tongue squamous cell carcinoma. J Clin Pathol. (2016) 69:742–4. 10.1136/jclinpath-2015-203599 27095085 PMC4975854

[26] Humphries HN Wickremesekera SK Marsh RW Brasch HD Mehrota S Tan ST. Characterization of cancer stem cells in colon adenocarcinoma metastasis to the liver. Front Surg. (2018) 4. 10.3389/fsurg.2017.00076 29404335 PMC5786574

[27] Cane R Kennedy-Smith A. Brasch HD, Savage S, Marsh RW, Itinteang T, Tan ST, Itinteang T. Characterization of cancer stem cells in renal clear cell carcinoma. J Stem Cell Regen Biol. (2019) 5:6–17.

[28] Ram R Brasch HD Dunne JC Davis PF Tan ST Itinteang T. The identification of three cancer stem cell subpopulations within moderately differentiated lip squamous cell carcinoma. Front Surg. (2017) 4. 10.3389/fsurg.2017.00012 28321397 PMC5337496

[29] Yu HH Featherston T Tan ST Chibnall AM Brasch HD Davis PF. Characterization of cancer stem cells in moderately differentiated buccal mucosal squamous cell carcinoma. Front Surg. (2016) 3:1. 10.3389/fsurg.2016.00046 27532037 PMC4970507

[30] Koh SP Brasch HD Jongh J de Itinteang T Tan ST. Cancer stem cell subpopulations in moderately differentiated head and neck cutaneous squamous cell carcinoma. Heliyon. (2019) 5. 10.1016/j.heliyon.2019.e02257 31463389 PMC6709152

[31] Yoganandarajah V Patel J Schaijik B van Bockett N Brasch HD Paterson E. Identification of cancer stem cell subpopulations in head and neck metastatic malignant melanoma. Cells. (2020) 9:324. 10.3390/cells9020324 32019273 PMC7072148

[32] Wickremesekera AC Brasch HD Lee VM Davis PF Woon K Johnson R. Expression of cancer stem cell markers in metastatic melanoma to the brain. J Clin Neurosci. (2019) 60:112–6. 10.1016/j.jocn.2018.10.068 30626524

[33] Siljee S Buchanan O Brasch HD Bockett N Patel J Paterson E. Cancer stem cells in metastatic head and neck cutaneous squamous cell carcinoma express components of the renin-angiotensin system. Cells. (2021) 10. 10.3390/cells10020243 33513805 PMC7910940

[34] Itinteang T Dunne JC Chibnall AM Brasch HD Davis PF Tan ST. Cancer stem cells in moderately differentiated oral tongue squamous cell carcinoma express components of the renin–angiotensin system. J Clin Pathol. (2016) 69:942–5. 10.1136/jclinpath-2016-203736 27371611 PMC5050289

[35] O'Rawe M Kilmister EJ Mantamadiotis T Kaye AH Tan ST Wickremesekera AC. The renin–angiotensin system in the tumor microenvironment of glioblastoma. Cancers. (2021) 13:4004. 10.3390/cancers13164004 34439159 PMC8392691

[36] On N Koh SP Brasch HD Dunne JC Armstrong JR Tan ST. Embryonic stem cell-like population in Dupuytren's disease expresses components of the renin-angiotensin system. Plast Reconstr Surg - Glob Open. (2017) 5. 10.1097/GOX.0000000000001422 28831359 PMC5548582

[37] Yakes WF Krauth L Ecklund J Swengle R Dreisbach JN Seibert CE. Ethanol endovascular management of brain arteriovenous malformations: Initial results. Neurosurgery. (1997) 40:1145–54. 10.1097/00006123-199706000-00005 9179886

[38] Nehme A Zouein FA Zayeri ZD Zibara K. An Update on the tissue renin angiotensin System and its role in physiology and pathology. J Cardiovasc Dev Dis. (2019) 6:14. 10.3390/jcdd6020014 30934934 PMC6617132

[39] Atlas SA. The renin-angiotensin aldosterone system: pathophysiological role and pharmacologic inhibition. J Manag Care Pharm. (2007) 13. 10.18553/jmcp.2007.13.s8-b.9 17970613 PMC10437584

[40] Dzau VJ Herrmann HC. Hormonal control of angiotensinogen production. Life Sci. (1982) 30:577–84. 10.1016/0024-3205(82)90272-7 7040893

[41] Afsar B Afsar RE Ertuglu LA Kuwabara M Ortiz A Covic A. Renin-angiotensin system and cancer: epidemiology, cell signaling, genetics and epigenetics. Clin Transl Oncol. (2021) 23:682–96. 10.1007/s12094-020-02488-3 32930920

[42] Ichihara A Yatabe MS. The (pro)renin receptor in health and disease. Nat Rev Nephrol. (2019) 15:693–712. 10.1038/s41581-019-0160-5 31164719

[43] Hsueh WA. Potential effects of renin activation on the regulation of renin production. Am J Physiol. (1984) 16. 10.1152/ajprenal.1984.247.2.F205 6087677

[44] Santos RAS Ferreira AJ Cristina Simões Silva A. Recent advances in the angiotensin-converting enzyme 2-angiotensin(1-7)-Mas axis. Exp Physiol Artic. (2008) 93:519–527. 10.1113/expphysiol.2008.042002 18310257

[45] Kim S Iwao H. Molecular and cellular mechanisms of angiotensin II-mediated cardiovascular and renal diseases. Pharmacol Rev. (2000) 52:11–34. 10699153

[46] George AJ Hannan RD Thomas WG. Unravelling the molecular complexity of GPCR-mediated EGFR transactivation using functional genomics approaches. FEBS J. (2013) 280:5258–68. 10.1111/febs.12509 23992425

[47] Jaffe IZ Mendelsohn ME. Angiotensin II and aldosterone regulate gene transcription via functional mineralocortocoid receptors in human coronary artery smooth muscle cells. Circ Res. (2005) 96:643–50. 10.1161/01.RES.0000159937.05502.d1 15718497

[48] Verhovez A Williams TA Morello F Monticone S Brizzi MF Dentelli P. Aldosterone does not modify gene expression in human endothelial cells. Horm Metab Res. (2012) 44:234–8. 10.1055/s-0031-1291272 22068811

[49] Steckelings UM Rompe F Kaschina E Namsolleck P Grzesiak A Funke-Kaiser H. The past, present and future of angiotensin II type 2 receptor stimulation. JRAAS - J Renin-Angiotensin-Aldosterone Syst. (2010) 11:67–73. 10.1177/1470320309347791 19861348

[50] Jackson L Eldahshan W Fagan SC Ergul A. Within the brain: the renin angiotensin system. Int J Mol Sci. (2018) 19:1–23. 10.3390/ijms19030876 29543776 PMC5877737

[51] Xu J Fan J Wu F Huang Q Guo M Lv Z. The ACE2/Angiotensin-(1–7)/Mas receptor axis: pleiotropic roles in cancer. Front Physiol. (2017) 8:276. 10.3389/fphys.2017.00276 28533754 PMC5420593

[52] Sobczuk P Szczylik C Porta C Czarnecka AM. Renin angiotensin system deregulation as renal cancer risk factor. Oncol Lett. (2017) 14:5059–68. 10.3892/ol.2017.6826 29098020 PMC5652144

[53] Geng YL Ding YJ Ni L Xu K Di Le VM Ji R. The role of angiotensin-(1-7) on acquired platinum resistance-induced angiogenesis in non-small cell lung cancer in vitro and in vivo. Neoplasma. (2021) 68:770–9. 10.4149/neo_2021_201213N1347 34034496

[54] Borghi C. SIIA Task Force, Rossi F, SIF Task Force. Role of the renin-angiotensin-aldosterone system and its pharmacological inhibitors in cardiovascular diseases: complex and critical issues High Blood Press. Cardiovasc Prev. (2015) 22:429–44. 10.1007/s40292-015-0120-5 26403596

[55] Rahman RMA van Schaijik B Brasch HD Marsh RW Wickremesekera AC Johnson R. Expression of cathepsins B, D, and G in WHO grade i meningioma. Front Surg. (2019) 6:6. 10.3389/fsurg.2019.00006 30949483 PMC6436525

[56] Ribeiro-Oliveira A Nogueira AI Pereira RM Boas WWV Santos RAS. dos, Silva ACS. The renin–angiotensin system and diabetes: an update. Vasc Health Risk Manag. (2008) 4:787. 10.2147/VHRM.S1905 19065996 PMC2597759

[57] Macconi D Remuzzi G Benigni A. Key fibrogenic mediators: old players. Renin-angiotensin system Kidney. Int Suppl. (2014) 4:58–64. 10.1038/kisup.2014.11 26312151 PMC4536968

[58] Hale TM. Persistent phenotypic shift in cardiac fibroblasts: impact of transient renin angiotensin system inhibition. J Mol Cell Cardiol. (2016) 93:125–32. 10.1016/j.yjmcc.2015.11.027 26631495

[59] Simões e Silva AC Teixeira MM. ACE inhibition, ACE2 and angiotensin-(1-7) axis in kidney and cardiac inflammation and fibrosis. Pharmacol Res. (2016) 107:154–162. 10.1016/j.phrs.2016.03.018 26995300

[60] Uhal BD Li X Piasecki CC Molina-Molina M. Angiotensin signalling in pulmonary fibrosis. Int J Biochem Cell Biol. (2012) 44:465–8. 10.1016/j.biocel.2011.11.019 22155301 PMC3288339

[61] Grace JA Herath CB Mak KY Burrell LM Angus PW. Update on new aspects of the renin-angiotensin system in liver disease: clinical implications and new therapeutic options. Clin Sci. (2012) 123:225–39. 10.1042/CS20120030 22548407

[62] Goossens GH. The renin-angiotensin system in the pathophysiology of type 2 diabetes. Obes Facts. (2012) 5:611–24. 10.1159/000342776 22986649

[63] Abdul-Hafez A Mohamed T Omar H Shemis M Uhal BD. The renin angiotensin system in liver and lung: impact and therapeutic potential in organ fibrosis. J lung, Pulm Respir Res. (2018) 5. 10.15406/jlprr.2018.05.00160 30175235 PMC6114139

[64] Haznedaroglu IC Beyazit Y. Local bone marrow renin-angiotensin system in primitive, definitive and neoplastic haematopoiesis. Clin Sci. (2013) 124:307–23. 10.1042/CS20120300 23157407

[65] Goker H Haznedaroglu IC Beyazit Y Aksu S Tuncer S Misirlioglu M. Local umbilical cord blood renin-angiotensin system. Ann Hematol. (2005) 84:277–81. 10.1007/s00277-004-0989-x 15645231

[66] Haznedaroglu IC Arici M Büyükaşik Y. A unifying hypothesis for the renin-angiotensin system and hematopoiesis: sticking the pieces together with the JAK-STAT pathway. Med Hypotheses. (2000) 54:80–3. 10.1054/mehy.1998.0830 10790731

[67] Casares MTG Iglesia SDL Perera M Lemes A Campo C Miguel JDGSM. Renin expression in hematological malignancies and its role in the regulation of hematopoiesis. Leuk Lymphoma. (2002) 43:2377–81. 10.1080/1042819021000040080 12613527

[68] George AJ Thomas WG Hannan RD. The renin–angiotensin system and cancer: old dog, new tricks. Nat Rev Cancer 2010 1011. (2010) 10:745–759. 10.1038/nrc2945 20966920

[69] Pinter M Jain RK. Targeting the renin-angiotensin system to improve cancer treatment: implications for immunotherapy. Sci Transl Med. (2017) 9:5616. 10.1126/scitranslmed.aan5616 28978752 PMC5928511

[70] Arneth B. Tumor microenvironment. Medicina. (2019) 56:15. 10.3390/medicina56010015 31906017 PMC7023392

[71] Hanahan D Weinberg RA. Hallmarks of cancer: the next generation. Cell. (2011) 144:646–74. 10.1016/j.cell.2011.02.013 21376230

[72] Lee B Laredo J. Classification: general overview. In: Vasc Malformations. (2021) p. 29–35. 10.1007/978-981-15-9762-6_4

[73] Müller-Wille R Wildgruber M Sadick M Wohlgemuth WA. Vascular anomalies (part II): interventional therapy of peripheral vascular malformations. Rofo. (2018) 190:927–37. 10.1055/s-0044-101266 29415296

[74] Itinteang T Tan ST Brasch HD Steel R Best HA Vishvanath A. Infantile haemangioma expresses embryonic stem cell markers. J Clin Pathol. (2012) 65:394–8. 10.1136/jclinpath-2011-200462 22447921

[75] Eady EK Brasch HD De Jongh J Marsh RW Tan ST Itinteang T. Expression of embryonic stem cell markers in microcystic lymphatic malformation. Lymphat Res Biol. (2019) 17:496–503. 10.1089/lrb.2018.0046 30901291

[76] Itinteang T Brasch HD Tan ST Day DJ. Expression of components of the renin–angiotensin system in proliferating infantile haemangioma may account for the propranolol-induced accelerated involution. J Plast Reconstr Aesthetic Surg. (2011) 64:759–65. 10.1016/j.bjps.2010.08.039 20870476

[77] Papali'i-Curtin JC Brasch HD van Schaijik B de Jongh J Marsh RW Tan ST. Expression of components of the renin-angiotensin system in pyogenic granuloma. Front Surg. (2019) 6:13. 10.3389/fsurg.2019.00013 31024924 PMC6465765

[78] Tan EMS Brasch HD Davis PF Itinteang T Tan ST. Embryonic stem cell-like population within venous malformation expresses the renin–angiotensin system. Plast Reconstr Surg Glob Open. (2019) 7. 10.1097/GOX.0000000000002170 31321175 PMC6554167

[79] Siljee S Keane E Marsh R Brasch HD Tan ST Itinteang T. Expression of the components of the renin–angiotensin system in venous malformation. Front Surg. (2016) 3:24. 10.3389/fsurg.2016.00024 27200356 PMC4853390

[80] Siljee SD Gower A Brasch HD Patel J Bockett N Itinteang T. Expression of angiotensin II receptor 2 in microcystic lymphatic malformation. J Vasc Anomalies. (2021) 2:e020. 10.1097/JOVA.000000000000002030901291

[81] Smithson SL Rademaker M Adams S Bade S Bekhor P Davidson S. Consensus statement for the treatment of infantile haemangiomas with propranolol. Australas J Dermatol. (2017) 58:155–9. 10.1111/ajd.12600 28251611

[82] Tan BH Leadbitter P Aburn N Tan ST. Steroid therapy for problematic proliferating haemangioma. N Z Med J. (2011) 124:57–65. 21475361

[83] Léauté-Labrèze C de la Roque ED Hubiche T Boralevi F Thambo J-B Taïeb A. Propranolol for severe hemangiomas of infancy. N Engl J Med. (2008) 358:2649–51. 10.1056/NEJMc0708819 18550886

[84] Bigorre M Van Kien AK Valette H. Beta-blocking agent for treatment ofinfantile hemangioma. Plast Reconstr Surg. (2009) 123. 10.1097/PRS.0b013e3181a3f435 19483538

[85] Koh SP Leadbitter P Smithers F Tan ST. β-blocker therapy for infantile haemangioma. Expert Rev Clin Pharmacol. (2020) 13:899–915. 10.1080/17512433.2020.1788938 32662682

[86] Itinteang T Withers AHJ Leadbitter P Day DJ Tan ST. Pharmacologic therapies for infantile hemangioma: is there a rational basis? Plast Reconstr Surg. (2011) 128:499–507. 10.1097/PRS.0b013e31821b63a0 21788841

[87] Sulzberger L Baillie R Itinteang T De Jong S Marsh R Leadbitter P. Serum levels of renin, angiotensin-converting enzyme and angiotensin II in patients treated by surgical excision, propranolol and captopril for problematic proliferating infantile haemangioma. J Plast Reconstr Aesthet Surg. (2016) 69:381–6. 10.1016/j.bjps.2015.10.020 26612192

[88] Xu D Teresa MO Shartava A Fowles TC Yang J Fink LM. Isolation, characterization, and in vitro propagation of infantile hemangioma stem cells and an *in vivo* mouse model. J Hematol Oncol. (2011) 4:54. 10.1186/1756-8722-4-54 22192404 PMC3259074

[89] Itinteang T Tan ST Brasch H Day DJ. Haemogenic endothelium in infantile haemangioma. J Clin Pathol. (2010) 63:982–6. 10.1136/jcp.2010.081257 20924092

[90] Itinteang T Tan ST Brasch H Day DJ. Primitive mesodermal cells with a neural crest stem cell phenotype predominate proliferating infantile haemangioma. J Clin Pathol. (2010) 63:771–6. 10.1136/jcp.2010.079368 20696686

[91] Itinteang T Vishvanath A Day DJ Tan ST. Mesenchymal stem cells in infantile haemangioma. J Clin Pathol. (2011) 64:232–6. 10.1136/jcp.2010.085209 21242328

[92] Itinteang T Tan ST Brasch HD Vishvanath A Day DJ. Primitive erythropoiesis in infantile haemangioma. Br J Dermatol. (2011) 164:1097–100. 10.1111/j.1365-2133.2010.10187.x 21518328

[93] Hirschi KK. Hemogenic endothelium during development and beyond. Blood. (2012) 119:4823. 10.1182/blood-2011-12-353466 22415753 PMC3367889

[94] Rivière JB Mirzaa GM O'Roak BJ Beddaoui M Alcantara D Conway RL. De novo germline and postzygotic mutations in AKT3, PIK3R2 and PIK3CA cause a spectrum of related megalencephaly syndromes. Nat Genet. (2012) 44:934–40. 10.1038/ng.2331 22729224 PMC3408813

[95] van Schaijik B Tan ST Marsh RW Itinteang T. Expression of (pro)renin receptor and its effect on endothelial cell proliferation in infantile hemangioma. Pediatr Res. (2019) 86:202–7. 10.1038/s41390-019-0430-8 31091531

[96] Sakoda M Ichihara A Kaneshiro Y Takemitsu T Nakazato Y Nabi AHMN. (Pro)renin receptor–mediated activation of mitogen-activated protein kinases in human vascular smooth muscle cells. Hypertens Res. (2007) 30:1139–46. 10.1291/hypres.30.1139 18250563

[97] Balakumar P Jagadeesh G. Potential cross-talk between (pro)renin receptors and Wnt/frizzled receptors in cardiovascular and renal disorders. Hypertens Res. (2011) 34:1161–70. 10.1038/hr.2011.113 21796133

[98] Itinteang T Marsh R Davis PF Tan ST. Angiotensin II causes cellular proliferation in infantile haemangioma via angiotensin II receptor 2 activation. J Clin Pathol. (2015) 68:346–50. 10.1136/jclinpath-2014-202794 25713419

[99] Dillon MJ Ryness JM. Plasma renin activity and aldosterone concentration in children. Br Med J. (1975) 4:316–9. 10.1136/bmj.4.5992.316 1192047 PMC1675185

[100] Fiselier TJW Lijnen P Monnens L van Munster P Jansen M Peer P. Levels of renin, angiotensin I and II, angiotensin-converting enzyme and aldosterone in infancy and childhood. Eur J Pediatr. (1983) 141:3–7. 10.1007/BF00445660 6315441

[101] Pipkin FB Smales OR O'Callaghan MJ. Renin and angiotensin levels in children. Arch Dis Child. (1981) 56:298–302. 10.1136/adc.56.4.298 7018406 PMC1627246

[102] Tan ST Itinteang T Day DJ O'Donnell C Mathy JA Leadbitter P. Treatment of infantile haemangioma with captopril. Br J Dermatol. (2012) 167:619–24. 10.1111/j.1365-2133.2012.11016.x 22533490

[103] Itinteang T Chudakova DA Dunne JC Davis PF Tan ST. Expression of cathepsins B, D, and G in infantile hemangioma. Front Surg. (2015) 2. 10.3389/fsurg.2015.00026 26137466 PMC4470331

[104] Tan EMS Chudakova DA Davis PF Brasch HD Itinteang T Tan ST. Characterisation of subpopulations of myeloid cells in infantile haemangioma. J Clin Pathol. (2015) 68:571–4. 10.1136/jclinpath-2014-202846 25834091

[105] Tan ST Wallis RA He Y Davis PF. Mast cells and hemangioma. Plast Reconstr Surg. (2004) 113:999–1011. 10.1097/01.PRS.0000105683.10752.A6 15108898

[106] Overman J Fontaine F Sears JW Moustaqil M Huang L Meurer M. R-propranolol is a small molecule inhibitor of the SOX18 transcription factor in a rare vascular syndrome and hemangioma. Elife. (2019) 8. 10.7554/eLife.43026 31358114 PMC6667216

[107] Sasaki M North PE Elsey J Bubley J Rao S Jung Y. Propranolol exhibits activity against hemangiomas independent of beta blockade. npj Precis Oncol. (2019) 3:1–9. 10.1038/s41698-019-0099-9 31701018 PMC6825155

[108] Durik M Pessôa BS Roks AJM. The renin-angiotensin system, bone marrow and progenitor cells. Clin Sci. (2012) 123:205–23. 10.1042/CS20110660 22548406

[109] Limaye N Kangas J Mendola A Godfraind C Schlögel MJ Helaers R. Somatic activating PIK3CA mutations cause venous malformation. Am J Hum Genet. (2015) 97:914–21. 10.1016/j.ajhg.2015.11.011 26637981 PMC4678782

[110] Couto JA Huang AY Konczyk DJ Goss JA Fishman SJ Mulliken JB. Somatic MAP2K1 mutations are associated with extracranial arteriovenous malformation. Am J Hum Genet. (2017) 100:546–54. 10.1016/j.ajhg.2017.01.018 28190454 PMC5339083

[111] Revencu N Boon LM Mendola A Cordisco MR Dubois J Clapuyt P. RASA1 mutations and associated phenotypes in 68 families with capillary malformation–arteriovenous malformation. Hum Mutat. (2013) 34:1632–41. 10.1002/humu.22431 24038909

[112] Mehrotra S Van Schaijik B Boyes K Bockett N Brasch HD Davis PF. Expression of cathepsins B, D, and G in microcystic lymphatic malformation. Lymphat Res Biol. (2021) 19:347–54. 10.1089/lrb.2020.0047 33337924

[113] Hansen L Brasch HD Paterson E Patel J Bockett N Davis PF. Expression of Cathepsins B, D, and G in extracranial arterio-venous malformation. Front Surg. (2021) 8:281. 10.3389/fsurg.2021.676871 34409065 PMC8367294

[114] Koh SP Brasch HD Patel J Bockett N Paterson E Davis PF. Expression of cathepsins B, D, and G in hypertrophic port-wine stain. J Vasc Anomalies. (2021) 2:e022. 10.1097/JOVA.0000000000000022

[115] Venot Q Blanc T Rabia SH Berteloot L Ladraa S Duong JP. Targeted therapy in patients with PIK3CA-related overgrowth syndrome. Nature. (2018) 558:540–6. 10.1038/s41586-018-0217-9 29899452 PMC7610773

[116] Al-Olabi L Polubothu S Dowsett K Andrews KA Stadnik P Joseph AP. Mosaic RAS/MAPK variants cause sporadic vascular malformations which respond to targeted therapy. J Clin Invest. (2018) 128:1496–508. 10.1016/j.jid.2018.03.765 29461977 PMC5873857

[117] Keppler-Noreuil KM Sapp JC Lindhurst MJ Darling TN Burton-Akright J Bagheri M. Pharmacodynamic study of miransertib in individuals with proteus syndrome. Am J Hum Genet. (2019) 104:484–91. 10.1016/j.ajhg.2019.01.015 30803705 PMC6407523

[118] Atkinson V Sandhu S Hospers G Long GV Aglietta M Ferrucci PF. Dabrafenib plus trametinib is effective in the treatment of BRAF V600-mutated metastatic melanoma patients: analysis of patients from the dabrafenib plus trametinib Named Patient Program (DESCRIBE II). Melanoma Res. (2020) 30:261–7. 10.1097/CMR.0000000000000654 31895752

[119] Subbiah V Meyer C Zinner R Meric-Bernstam F Zahurak ML O'Connor A. Phase Ib/II study of the safety and efficacy of combination therapy with multikinase vegf inhibitor pazopanib and mek inhibitor trametinib in advanced soft tissue sarcoma. Clin Cancer Res. (2017) 23:4027–34. 10.1158/1078-0432.CCR-17-0272 28377484 PMC5754188

[120] Kilmister EJ Robinson B De Tommasi C. Treatment of BRAF V600E mutated ganglioglioma of the third ventricle with dabrafenib. Surg Neurol Int. (2021) 12:529. 10.25259/SNI_788_2021 34754579 PMC8571360

[121] Meng J Peng H Dai B Guo W Wang L Ji L. High level of AKT activity is associated with resistance to MEK inhibitor AZD6244 (ARRY-142886). Cancer Biol Ther. (2009) 8:2073–80. 10.4161/cbt.8.21.9844 19783898 PMC2835993

[122] Wee S Jagani Z Kay XX Loo A Dorsch M Yao YM. PI3K pathway activation mediates resistance to MEK inhibitors in KRAS mutant cancers. Cancer Res. (2009) 69:4286–93. 10.1158/0008-5472.CAN-08-4765 19401449

[123] Kobayashi S Kishimoto T Kamata S Otsuka M Miyazaki M Ishikura H. Rapamycin, a specific inhibitor of the mammalian target of rapamycin, suppresses lymphangiogenesis and lymphatic metastasis. Cancer Sci. (2007) 98:726–33. 10.1111/j.1349-7006.2007.00439.x 17425689 PMC11158643

[124] Xue Q Nagy JA Manseau EJ Phung TL Dvorak HF Benjamin LE. Rapamycin inhibition of the AKT/mTOR pathway blocks select stages of VEGF-a164-driven angiogenesis, in part by blocking S6kinase. Arterioscler Thromb Vasc Biol. (2009) 29:1172–8. 10.1161/ATVBAHA.109.185918 19443844 PMC2756965

[125] Kasap B. Sirolimus in pediatric renal transplantation. Pediatr Transplant. (2011) 15:673–85. 10.1111/j.1399-3046.2011.01575.x 22004542

[126] Freixo C Ferreira V Martins J Almeida R Caldeira D Rosa M. Efficacy and safety of sirolimus in the treatment of vascular anomalies: a systematic review. J Vasc Surg. (2020) 71:318–27. 10.1016/j.jvs.2019.06.217 31676179

[127] Parker VER Keppler-Noreuil KM Faivre L Luu M Oden NL De Silva L. Safety and efficacy of low-dose sirolimus in the PIK3CA-related overgrowth spectrum. Genet Med. (2018) 21:1189–1198. 10.1038/s41436-018-0297-9 30270358 PMC6752269

[128] Hammill AM Wentzel M Gupta A Nelson S Lucky A Elluru R. Sirolimus for the treatment of complicated vascular anomalies in children. Pediatr Blood Cancer. (2011) 57:1018–24. 10.1002/pbc.23124 21445948

[129] Aarts M Georgilis A Beniazza M Beolchi P Banito A Carroll T. Coupling shRNA screens with single-cell RNA-seq identifies a dual role for mTOR in reprogramming-induced senescence. Genes Dev. (2017) 31:2085–98. 10.1101/gad.297796.117 29138277 PMC5733499

[130] Lee SJ Kang KW Kim JH Lee BH Jung JH Park Y. CXCR2 Ligands and mTOR activation enhance reprogramming of human somatic cells to pluripotent stem cells. Stem Cells Dev. (2020) 29:119–32. 10.1089/scd.2019.0188 31808362

[131] Bulut-Karslioglu A Biechele S Jin H MacRae TA Hejna M Gertsenstein M. Inhibition of mTOR induces a paused pluripotent state. Nat. (2016) 540:119–23. 10.1038/nature20578 27880763 PMC5143278

[132] Biserova K Jakovlevs A Uljanovs R Strumfa I. Cells cancer stem cells: significance in origin, pathogenesis and treatment of glioblastoma. Cells. (2021) 10:621. 10.3390/cells10030621 33799798 PMC8000844

[133] Akbarzadeh M Maroufi NF Tazehkand AP Akbarzadeh M Bastani S Safdari R. Current approaches in identification and isolation of cancer stem cells. J Cell Physiol. (2019) 234:14759–72. 10.1002/jcp.28271 30741412

[134] Schatton T Frank MH. Antitumor immunity and cancer stem cells. Ann N Y Acad Sci. (2009) 1176:154. 10.1111/j.1749-6632.2009.04568.x 19796244 PMC2893543

[135] Afify SM Seno M. Conversion of stem cells to cancer stem cells: undercurrent of cancer initiation. Cancers. (2019) 11:345. 10.3390/cancers11030345 30862050 PMC6468812

[136] Crabtree JS Miele L. Breast cancer stem cells. Biomed. (2018) 6:77. 10.3390/biomedicines6030077 30018256 PMC6163894

[137] Lang SH Frame FM Collins AT. Prostate cancer stem cells. J Pathol. (2009) 217:299–306. 10.1002/path.2478 19040209 PMC2673349

[138] Qian CN Mei Y Zhang J. Cancer metastasis: issues and challenges. Chin J Cancer. (2017) 36:1–4. 10.1186/s40880-017-0206-7 28372569 PMC5379757

[139] Nimmakayala RK Batra SK Ponnusamy MP. Unraveling the journey of cancer stem cells from origin to metastasis. In: Elsevier B.V (2019). p. 50–63. 10.1016/j.bbcan.2018.10.006 30419314 PMC6347501

[140] Massagué J Obenauf AC. Metastatic colonization by circulating tumour cells. Nature. (2016) 529:298–306. 10.1038/nature17038 26791720 PMC5029466

[141] Luzzi KJ MacDonald IC Schmidt EE Kerkvliet N Morris VL Chambers AF. Multistep nature of metastatic inefficiency: dormancy of solitary cells after successful extravasation and limited survival of early micrometastases. Am J Pathol. (1998) 153:865–73. 9736035 10.1016/S0002-9440(10)65628-3 PMC1853000

[142] Abbaszadegan MR Bagheri V Razavi MS Momtazi AA Sahebkar A Gholamin M. Isolation, identification, and characterization of cancer stem cells: a review. J Cell Physiol. (2017) 232:2008–18. 10.1002/jcp.25759 28019667

[143] Liao WT Ye YP Deng YJ Bian XW Ding YQ. Metastatic cancer stem cells: from the concept to therapeutics. Am J Stem Cells. (2014) 3:46. 25232505 PMC4163604

[144] Gammon L Biddle A Heywood HK Johannessen AC Mackenzie IC. Sub-sets of cancer stem cells differ intrinsically in their patterns of oxygen metabolism. PLoS ONE. (2013) 8:e62493. 10.1371/journal.pone.0062493 23638097 PMC3640080

[145] Gupta PB Chaffer CL Weinberg RA. Cancer stem cells: mirage or reality? Nat Med. (2009) 15:1010–2. 10.1038/nm0909-1010 19734877

[146] Reichert M Bakir B Moreira L Pitarresi JR Feldmann K Simon L. Regulation of epithelial plasticity determines metastatic organotropism in pancreatic cancer. Dev Cell. (2018) 45:696-711.e8. 10.1016/j.devcel.2018.05.025 29920275 PMC6011231

[147] Sampieri K Fodde R. Cancer stem cells and metastasis. Semin Cancer Biol. (2012) 22:187–93. 10.1016/j.semcancer.2012.03.002 22774232

[148] Hermann PC Huber SL Herrler T Aicher A Ellwart JW Guba M. Distinct populations of cancer stem cells determine tumor growth and metastatic activity in human pancreatic cancer. Cell Stem Cell. (2007) 1:313–23. 10.1016/j.stem.2007.06.002 18371365

[149] Brougham NDL Dennett ER Tan ST. Changing incidence of non-melanoma skin cancer in New Zealand. ANZ J Surg. (2011) 81:633–6. 10.1111/j.1445-2197.2010.05583.x 22295395

[150] Brougham NDL Dennett ER Cameron R Tan ST. The incidence of metastasis from cutaneous squamous cell carcinoma and the impact of its risk factors. J Surg Oncol. (2012) 106:811–5. 10.1002/jso.23155 22592943

[151] Brougham NDL Tan ST. The incidence and risk factors of metastasis for cutaneous squamous cell carcinoma–implications on the T-classification system. J Surg Oncol. (2014) 110:876–82. 10.1002/jso.23731 25088537

[152] Rudolph R Zelac DE. Squamous cell carcinoma of the skin. Plast Reconstr Surg. (2004) 114. 10.1097/01.PRS.0000138243.45735.8A 15509920

[153] Kovach BT Stasko T. Skin cancer after transplantation. Transplant Rev. (2009) 23:178–89. 10.1016/j.trre.2009.02.004 19345080

[154] Ch'ng S Maitra A Allison RS Chaplin JM Gregor RT Lea R. Parotid and cervical nodal status predict prognosis for patients with head and neck metastatic cutaneous squamous cell carcinoma. J Surg Oncol. (2008) 98:101–105. 10.1002/jso.21092 18523982

[155] Yuan S Zhang P Wen L Jia S Wu Y Zhang Z. MiR-22 promotes stem cell traits via activating Wnt/β-catenin signaling in cutaneous squamous cell carcinoma. Oncogene. (2021) 40:5799–813. 10.1038/s41388-021-01973-5 34345013 PMC8484012

[156] Nallaiah S Lee VMY Brasch HD de Jongh J van Schaijik B. Cancer stem cells within moderately differentiated head and neck cutaneous squamous cell carcinoma express components of the renin-angiotensin system. J Plast Reconstr Aesthetic Surg. (2019) 72:1484–93. 10.1016/j.bjps.2018.11.013 30528285

[157] Siegel RL Miller KD Jemal A. Cancer statistics, 2020. CA Cancer J Clin. (2020) 70:7–30. 10.3322/caac.21590 31912902

[158] Perera E Gnaneswaran N Jennens R Sinclair R. Malignant melanoma. Healthc. (2013) 2:1–19. 10.3390/healthcare2010001 27429256 PMC4934490

[159] White RR Stanley WE Johnson JL Tyler DS Seigler HF. Long-term survival in 2,505 patients with melanoma with regional lymph node metastasis. Ann Surg. (2002) 235:879–87. 10.1097/00000658-200206000-00017 12035046 PMC1422519

[160] Panje WR Moran WJ. Melanoma of the upper aerodigestive tract: A review of 21 cases. Head Neck Surg. (1986) 8:309–12. 10.1002/hed.2890080412 3744859

[161] Trapp TK Fu YS Calcaterra TC. Melanoma of the nasal and paranasal sinus mucosa. Arch Otolaryngol Neck Surg. (1987) 113:1086–9. 10.1001/archotol.1987.01860100064023 3620131

[162] Stark MS Woods SL Gartside MG Bonazzi VF Dutton-Regester K Aoude LG. Frequent somatic mutations in MAP3K5 and MAP3K9 in metastatic melanoma identified by exome sequencing. Nat Genet. (2011) 44:165–169. 10.1038/ng.1041 22197930 PMC3267896

[163] Flaherty K. Inhibition of mutated, activated BRAF in metastatic melanoma. N Engl J Med. (2010) 363:809–19. 10.1056/NEJMoa1002011 20818844 PMC3724529

[164] Chapman P. Improved survival with vemurafenib in melanoma with BRAF V600E mutation. N Engl J Med. (2011) 364:2507–16. 10.1056/NEJMoa1103782 21639808 PMC3549296

[165] Hauschild A. Dabrafenib in BRAF-mutated metastatic melanoma: a multicentre, open-label, phase 3 randomised controlled trial. Lancet. (2012) 380:358–65. 10.1016/S0140-6736(12)60868-X 22735384

[166] Weiss SA Wolchok JD Sznol M. Immunotherapy of melanoma: facts and hopes. Clin Cancer Res. (2019) 25:5191–201. 10.1158/1078-0432.CCR-18-1550 30923036 PMC6726509

[167] Chang PY Huang WY Lin CL Huang TC Wu YY Chen JH. Propranolol reduces cancer risk: a population-based cohort study. Medicine. (2015) 94:e1097. 10.1097/MD.0000000000001097 26166098 PMC4504645

[168] Hiller JG Cole SW Crone EM Byrne DJ Shackleford DM Pang JMB. Preoperative β-blockade with propranolol reduces biomarkers of metastasis in breast cancer: a phase II randomized trial. Clin Cancer Res. (2020) 26:1803–11. 10.1158/1078-0432.CCR-19-2641 31754048

[169] Cole SW Sood AK. Molecular pathways: beta-adrenergic signaling in cancer. Clin Cancer Res. (2012) 18:1201–6. 10.1158/1078-0432.CCR-11-0641 22186256 PMC3294063

[170] Calvani M Bruno G Dabraio A Subbiani A Bianchini F Fontani F. β3- Adrenoreceptor blockade induces stem cells differentiation in melanoma microenvironment. Int J Mol Sci. (2020) 21:1420. 10.3390/ijms21041420 32093135 PMC7073111

[171] Siljee S Pilkington T Brasch HD Bockett N Patel J Paterson E. Cancer stem cells in head and neck metastatic malignant melanoma express components of the renin-angiotensin system. Life. (2020) 10:268. 10.3390/life10110268 33147716 PMC7694034

[172] Wickremesekera AC Brasch HD Lee VM Davis PF Parker A Koeck H. Cancer stem cell subpopulations in metastatic melanoma to the brain express components of the renin-angiotensin system. J Cancer Metastasis Treat. (2019) 5:62. 10.20517/2394-4722.2019.009

[173] Ishikane S Hosoda H Nojiri T Tokudome T Mizutani T Miura K. Angiotensin II promotes pulmonary metastasis of melanoma through the activation of adhesion molecules in vascular endothelial cells. Biochem Pharmacol. (2018) 154:136–47. 10.1016/j.bcp.2018.04.012 29674000

[174] Nakamura K Kiniwa Y Okuyama R. CCL5 production by fibroblasts through a local renin–angiotensin system in malignant melanoma affects tumor immune responses. J Cancer Res Clin Oncol. (2021) 147:1993–2001. 10.1007/s00432-021-03612-8 33770254 PMC11801846

[175] Renziehausen A Wang H Rao B Weir L Nigro C Lo. The renin angiotensin system (RAS) mediates bifunctional growth regulation in melanoma and is a novel target for therapeutic intervention. Oncogene. (2019) 38:2320–36. 10.1038/s41388-018-0563-y 30478450

[176] Lever AF Hole DJ Gillis CR McCallum IR McInnes GT MacKinnon PL. Do inhibitors of angiotensin-I-converting enzyme protect against risk of cancer? Lancet. (1998) 352:179–84. 10.1016/S0140-6736(98)03228-0 9683206

[177] Sun H Li Tao Zhuang R Cai W Zheung Y. Do renin-angiotensin system inhibitors influence the recurrence, metastasis, and survival in cancer patients?: evidence from a meta-analysis including 55 studies. Medicine. (2017) 96. 10.1097/MD.0000000000006394 28353566 PMC5380250

[178] Shen J Haung Y-M Wang M Hong X-Z Song X-N Zou X. Renin-angiotensin system blockade for the risk of cancer and death. J Renin Angiotensin Aldosterone Syst. (2016) 17. 10.1177/1470320316656679 27402638 PMC5843874

[179] Zhou Q Chen DS Xin L Zhou LQ Zhang HT Liu L. The renin-angiotensin system blockers and survival in digestive system malignancies: a systematic review and meta-analysis. Med. (2020) 99. 10.1097/MD.0000000000019075 32049809 PMC7035076

[180] Li XY Sun JF Hu SQ. The renin-angiotensin system blockers as adjunctive therapy for cancer: a meta-analysis of survival outcome. Eur Rev Med Pharmacol Sci. (2017) 21:1375–83. 28387887

[181] Sipahi I Debanne SM Rowland DY Simon DI Fang JC. Angiotensin-receptor blockade and risk of cancer: meta-analysis of randomised controlled trials. Lancet Oncol. (2010) 11:627–36. 10.1016/S1470-2045(10)70106-6 20542468 PMC4070221

[182] Friis S Sørensen HT Mellemkjaer L Mclaughlin JK Nielsen GL Blot WJ. Angiotensin-converting enzyme inhibitors and the risk of cancer a population-based cohort study in denmark. Cancer. (2001) 92:2462–70. 10.1002/1097-0142(20011101)92:9<2462::AID-CNCR1596>3.0.CO;2-L 11745304

[183] Teo KK. Effects of telmisartan, irbesartan, valsartan, candesartan, and losartan on cancers in 15 trials enrolling 138 769 individuals. J Hypertens. (2011) 29:623–35. 10.1097/HJH.0b013e328344a7de 21358417

[184] Ishikane S Takahashi-Yanaga F. The role of angiotensin II in cancer metastasis: Potential of renin-angiotensin system blockade as a treatment for cancer metastasis. Biochem Pharmacol. (2018) 151:96–103. 10.1016/j.bcp.2018.03.008 29534876

[185] Medeiros R Vasconcelos A Costa S Pinto D Lobo F Morais A. Linkage of angiotensin I-converting enzyme gene insertion/deletion polymorphism to the progression of human prostate cancer. J Pathol. (2004) 202:330–5. 10.1002/path.1529 14991898

[186] Munro MJ Peng L Wickremesekera SK Tan ST. Colon adenocarcinoma-derived cells possessing stem cell function can be modulated using renin-angiotensin system inhibitors. PLoS ONE. (2021) 16:e0256280. 10.1371/journal.pone.0256280 34428252 PMC8384197

[187] O'Rawe M Wickremesekera AC Pandey R Young D Sim D FitzJohn T. Treatment of glioblastoma with re-purposed renin-angiotensin system modulators: Results of a phase I clinical trial. J Clin Neurosci. (2022) 95:48–54. 10.1016/j.jocn.2021.11.023 34929651

[188] Kast RE Karpel-Massler G Halatsch ME. CUSP9^*^ treatment protocol for recurrent glioblastoma: aprepitant, artesunate, auranofin, captopril, celecoxib, disulfiram, itraconazole, ritonavir, sertraline augmenting continuous low dose temozolomide. Oncotarget. (2014) 5:8052. 10.18632/oncotarget.2408 25211298 PMC4226667

[189] Zhang Y Kong J Deb DK Chang A Li YC. Vitamin D receptor attenuates renal fibrosis by suppressing the renin-angiotensin system. J Am Soc Nephrol. (2010) 21:966–73. 10.1681/ASN.2009080872 20378820 PMC2900963

[190] Shih B Bayat A. Genetics of keloid scarring. Arch Dermatol Res. (2010) 302:319–39. 10.1007/s00403-009-1014-y 20130896

[191] Huang C Murphy GF Akaishi S Ogawa R. Keloids and hypertrophic scars: update and future directions. Plast Reconstr Surg Glob Open. (2013) 1:e25. 10.1097/GOX.0b013e31829c4597 25289219 PMC4173836

[192] Paterson C Lee VMY Brasch HD van Schaijik B Marsh R Tan ST. Expression of cathepsins B, D, and G by the embryonic stem cell-like population within human keloid tissues and keloid-derived primary cell lines. Plast Reconstr Surg. (2019) 144:1338–49. 10.1097/PRS.0000000000006275 31764649

[193] Zhang Q Yamaza T Kelly AP Shi S Wang S Brown J. Tumor-like stem cells derived from human keloid are governed by the inflammatory niche driven by IL-17/IL-6 axis. PLoS ONE. (2009) 4. 10.1371/journal.pone.0007798 19907660 PMC2771422

[194] Humphries H Brasch HD van Schaijik B Tan ST Itinteang T. Expression of components of the renin-angiotensin system by the embryonic stem cell-like population within keloid lesions. Plast Reconstr Surg. (2019) 144:372–84. 10.1097/PRS.0000000000005867 31348346

[195] Ibrahim MM. RAS inhibition in hypertension. J Hum Hypertens. (2006) 20:101–8. 10.1038/sj.jhh.1001960 16397519

[196] Peters H Border WA Noble NA. Targeting TGF-beta overexpression in renal disease: maximizing the antifibrotic action of angiotensin II blockade. Kidney Int. (1998) 54:1570–80. 10.1046/j.1523-1755.1998.00164.x 9844133

[197] Kaschina E Unger T. Angiotensin AT1/AT2 receptors: regulation, signalling and function. Blood Press. (2003) 12:70–88. 10.1080/08037050310001057 12797627

[198] Hall LM Kimlin MG Aronov PA Hammock BD Slusser JR Woodhouse LR. Vitamin D intake needed to maintain target serum 25-hydroxyvitamin S concentrations in participants with low sun exposure and dark skin pigmentation is substantially higher than current recommendations. J Nutr. (2010) 140:542–50. 10.3945/jn.109.115253 20053937 PMC2821886

[199] Shi Y Liu T Yao L Xing Y Zhao X Fu J. Chronic vitamin D deficiency induces lung fibrosis through activation of the renin-angiotensin system. Sci Reports. (2017) 7:1–10. 10.1038/s41598-017-03474-6 28607392 PMC5468249

[200] Xiong M Gong J Liu Y Xiang R Tan X. Loss of vitamin D receptor in chronic kidney disease: a potential mechanism linking inflammation to epithelial-to-mesenchymal transition. Am J Physiol Renal Physiol. (2012) 303:F1107–15. 10.1152/ajprenal.00151.2012 22791341

[201] Murphy AM Wong AL Bezuhly M. Modulation of angiotensin II signaling in the prevention of fibrosis. Fibrogenes Tissue Repair. (2015) 8:1–7. 10.1186/s13069-015-0023-z 25949522 PMC4422447

[202] Tang R Li Q Lv L Dai H Zheng M Ma K. Angiotensin II mediates the high-glucose-induced endothelial-to-mesenchymal transition in human aortic endothelial cells. Cardiovasc Diabetol. (2010) 9:31. 10.1186/1475-2840-9-31 20663195 PMC2920267

[203] Louw L. Keloids in rural black South Africans. Part 1: general overview and essential fatty acid hypotheses for keloid formation and prevention. Prostaglandins Leukot Essent Fatty Acids. (2000) 63:237–45. 10.1054/plef.2000.0207 11090249

[204] Holick MF Maclaughlin JA Clark MB Holick SA Potts JT Anderson RR. Photosynthesis of previtamin D3 in human skin and the physiologic consequences. Science. (1980) 210:203–5. 10.1126/science.6251551 6251551

[205] Arima J Huang C Rosner B Akaishi S Ogawa R. Hypertension: a systemic key to understanding local keloid severity. Wound Repair Regen. (2015) 23:213–21. 10.1111/wrr.12277 25728259

[206] Bivona G Agnello L Ciaccio M. The immunological implication of the new vitamin D metabolism. Cent J Immunol. (2018) 43:331. 10.5114/ceji.2018.80053 30588177 PMC6305614

[207] Webb AR Decosta BR Holick MF. Sunlight regulates the cutaneous production of vitamin d3 by causing its photodegradation. J Clin Endocrinol Metab. (1989) 68:882–7. 10.1210/jcem-68-5-882 2541158

[208] Haussler MR Whitfield GK Kaneko I Haussler CA Hsieh D Hsieh JC. Molecular mechanisms of vitamin D action. Calcif Tissue Int. (2012) 92:77–98. 10.1007/s00223-012-9619-0 22782502

[209] Bikle D. Nonclassic actions of vitamin D. J Clin Endocrinol Metab. (2009) 94:26–34. 10.1210/jc.2008-1454 18854395 PMC2630868

[210] Haussler MR Jurutka PW Mizwicki M Norman AW. Vitamin D receptor (VDR)-mediated actions of 1α,25(OH)_2_vitamin D3: genomic and non-genomic mechanisms. Best Pract Res Clin Endocrinol Metab. (2011) 25:543–59. 10.1016/j.beem.2011.05.010 21872797

[211] Ricca C Aillon A Bergandi L Alotto D Castagnoli C Silvagno F. Vitamin D receptor is necessary for mitochondrial function and cell health. Int J Mol Sci. (2018) 19:1672. 10.3390/ijms19061672 29874855 PMC6032156

[212] Rochel N Molnár F. Structural aspects of Vitamin D endocrinology. Mol Cell Endocrinol. (2017) 453:22–35. 10.1016/j.mce.2017.02.046 28257826

[213] Hindocha S Iqbal SA Farhatullah S Paus R Bayat A. Characterization of stem cells in Dupuytren's disease. Br J Surg. (2011) 98:308–15. 10.1002/bjs.7307 21104823

[214] Hindocha S McGrouther DA Bayat A. Epidemiological evaluation of Dupuytren's disease incidence and prevalence rates in relation to etiology. Hand (N Y). (2009) 4:256–69. 10.1007/s11552-008-9160-9 19145463 PMC2724613

[215] Armstrong JR Hurren JS Logan AM. Dermofasciectomy in the management of Dupuytren's disease. J Bone Joint Surg Br. (2000) 82:90–4. 10.1302/0301-620X.82B1.0820090 10697321

[216] Hurst LC Badalamente MA Hentz VR Hotchkiss RN Kaplan FTD Meals RA. Injectable collagenase clostridium histolyticum for Dupuytren's contracture. N Engl J Med. (2009) 361:968–79. 10.1056/NEJMoa0810866 19726771

[217] Iqbal SA Manning C Syed F Kolluru V Hayton M Watson S. Identification of mesenchymal stem cells in perinodular fat and skin in dupuytren's disease: a potential source of myofibroblasts with implications for pathogenesis and therapy. Stem Cells Dev. (2012) 21:609–22. 10.1089/scd.2011.0140 21612554 PMC3280606

[218] Stephen C Touil L Vaiude P Singh J McKirdy S. Angiotensin receptors in Dupuytren's disease: a target for pharmacological treatment? J Plast Surg Hand Surg. (2018) 52:37–9. 10.1080/2000656X.2017.1319846 28486091

[219] Chong SG Sato S Kolb M Gauldie J. Fibrocytes and fibroblasts—Where are we now. Int J Biochem Cell Biol. (2019) 116:105595. 10.1016/j.biocel.2019.105595 31473260

[220] Tan K Brasch HD van Schaijik B Armstrong JR Marsh RW Davis PF. Expression and localization of cathepsins B, D, and G in Dupuytren's disease. Plast Reconstr Surg. Glob Open. (2018) 6. 10.1097/GOX.0000000000001686 29616179 PMC5865920

[221] Herzog EL Bucala R. Fibrocytes in health and disease. Exp Hematol. (2010) 38:548–56. 10.1016/j.exphem.2010.03.004 20303382 PMC3136351

[222] Hedayatyanfard K Ziai SA Niazi F Habibi I Habibi B Moravvej H. Losartan ointment relieves hypertrophic scars and keloid: A pilot study. Wound Repair Regen. (2018) 26:340–3. 10.1111/wrr.12648 30099811

[223] Ardekani GS Aghaie S Nemati MH Handjani F Kasraee B. Treatment of a postburn keloid scar with topical captopril: report of the first case. Plast Reconstr Surg. (2009) 123. 10.1097/PRS.0b013e31819a34db 19319029

[224] Iannello S Milazzo P Bordonaro F Belfiore F. Low-dose enalapril in the treatment of surgical cutaneous hypertrophic scar and keloid - two case reports and literature review. Medscape Gen Med. (2006) 8:60. 17415337 PMC1868346

[225] Wegman-Ostrosky T Soto-Reyes E Vidal- Millán S Sánchez-Corona J. The renin-angiotensin system meets the hallmarks of cancer. J Renin Angiotensin Aldosterone Syst. (2015) 16:227–33. 10.1177/1470320313496858 23934336

[226] Zhang Z Zhou L Xie N Nice EC Zhang T Cui Y. Overcoming cancer therapeutic bottleneck by drug repurposing. Signal Transduct Target Ther. (2020) 5:1–25. 10.1038/s41392-020-00213-8 32616710 PMC7331117

